# A high-quality, haplotype-phased genome reconstruction reveals unexpected haplotype diversity in a pearl oyster

**DOI:** 10.1093/dnares/dsac035

**Published:** 2022-11-10

**Authors:** Takeshi Takeuchi, Yoshihiko Suzuki, Shugo Watabe, Kiyohito Nagai, Tetsuji Masaoka, Manabu Fujie, Mayumi Kawamitsu, Noriyuki Satoh, Eugene W Myers

**Affiliations:** Marine Genomics Unit, Okinawa Institute of Science and Technology Graduate University, Onna, Okinawa, Japan; Algorithms for Eco and Evo Genomics Unit, Okinawa Institute of Science and Technology Graduate University, Onna, Okinawa, Japan; Kitasato University School of Marine Biosciences, Sagamihara, Kanagawa, Japan; Pearl Research Institute, K. MIKIMOTO & CO., LTD, Shima, Mie, Japan; Aquatic Breeding Division, Aquaculture Research Department, Fisheries Technology Institute, Japan Fisheries Research and Education Agency, Tamaki, Mie, Japan; DNA Sequencing Section, Okinawa Institute of Science and Technology Graduate University, Onna, Okinawa, Japan; DNA Sequencing Section, Okinawa Institute of Science and Technology Graduate University, Onna, Okinawa, Japan; Marine Genomics Unit, Okinawa Institute of Science and Technology Graduate University, Onna, Okinawa, Japan; Algorithms for Eco and Evo Genomics Unit, Okinawa Institute of Science and Technology Graduate University, Onna, Okinawa, Japan; Max Planck Institute of Molecular Cell Biology and Genetics, Dresden, Germany; Center for Systems Biology Dresden, Dresden, Germany

**Keywords:** Mollusca, pearl oyster, haplotype-phased genome assembly, immunity, aquaculture

## Abstract

Homologous chromosomes in the diploid genome are thought to contain equivalent genetic information, but this common concept has not been fully verified in animal genomes with high heterozygosity. Here we report a near-complete, haplotype-phased, genome assembly of the pearl oyster, *Pinctada fucata*, using hi-fidelity (HiFi) long reads and chromosome conformation capture data. This assembly includes 14 pairs of long scaffolds (>38 Mb) corresponding to chromosomes (2*n* = 28). The accuracy of the assembly, as measured by an analysis of *k*-mers, is estimated to be 99.99997%. Moreover, the haplotypes contain 95.2% and 95.9%, respectively, complete and single-copy BUSCO genes, demonstrating the high quality of the assembly. Transposons comprise 53.3% of the assembly and are a major contributor to structural variations. Despite overall collinearity between haplotypes, one of the chromosomal scaffolds contains megabase-scale non-syntenic regions, which necessarily have never been detected and resolved in conventional haplotype-merged assemblies. These regions encode expanded gene families of NACHT, DZIP3/hRUL138-like HEPN, and immunoglobulin domains, multiplying the immunity gene repertoire, which we hypothesize is important for the innate immune capability of pearl oysters. The pearl oyster genome provides insight into remarkable haplotype diversity in animals.

## 1. Introduction

It is fundamental to construct a reference genome assembly as a platform to investigate the biological features of living organisms. To produce a genome assembly of a diploid organism, parental haplotypes are often collapsed to consensus sequences. This haplotype-merged assembly contains a complete set of genetic information vital for the organism, providing that parental haplotypes are genetically equivalent. This premise is valid for inbred model organisms with nearly identical haplotypes or species with low heterozygosity. In contrast, a haplotype-merged assembly of a highly heterozygous genome can lose a substantial amount of genetic variations maintained in only one of the parental haplotypes, resulting in a limited view of genetic diversity. This issue has been overlooked despite the recent rapid accumulation of genome assemblies of wild, non-model organisms that show high heterozygosity.

In general, marine invertebrates, including molluscs, have highly heterozygotic genomes. This is possibly due in part to their large population sizes and enormous fecundity.^1,2^ Recently, 12 chromosome-scale genome assemblies of molluscs have been reported using long reads and chromatin conformation capture data.^3–14^ All of these studies produced haplotype-merged assemblies, which are mosaics of sequences derived from chromosome pairs. As a result, these assemblies lose some of the genetic information in a diploid genome and can deviate notably from the true haploid genome sequence considering the high heterozygosity. It is important to establish a haplotype-phased genome assembly to thoroughly understand the genetic variation between haplotypes that characterizes molluscan genomes.

One of the major findings of molluscan genomics was remarkable gene family expansions related to immune recognition in bivalves.^15–18^ Bivalves are the second-largest group after the Gastropoda in the Phylum Mollusca, representing a wide range of habitats in aquatic environments from the intertidal zone to the deep seafloor. As they adopt filter-feeding and sedentary lifestyles in various habitats, lineage-specific gene expansions of the immune system may be essential to their ecological success to protect them from diverse microbial challenges. Recently, it was proposed that immunity and the stress-response gene repertoire are not consistent among individuals in the Mediterranean mussel, *Mytilus galloprovincialis*, demonstrating that genome diversity is linked to their evolutionary success.^19^ To test whether genome diversity is present within a species, it is essential to establish a haplotype-phased reference genome assembly.

The pearl oyster, *Pinctada fucata* (order Bivalvia, phylum Mollusca), receives special attention from researchers because of its value in the fishery industry for pearl production. Pearls are one of the most beautiful jewels, largely produced in Japan since technical innovations in their cultivation in the early 20th century.^20^ For the last decade, natural conditions at sites of pearl oyster fisheries have become less hospitable for reasons as yet not understood. In the 1990s in Japan, mass mortality induced by infectious diseases reduced pearl production to about one third over the course of a decade.^21,22^ Recently, fisheries have suffered disease outbreaks in juvenile and adult pearl oysters that may be related to both pathogens and increased seawater temperatures.^23,24^

In 2012, we published a draft genome of *P. fucata* (version 1.0), which was one of the first molluscan draft genomes.^25^ Subsequently, we improved the draft assembly (version 2.0) by producing more sequence data and applying a duplication removal strategy to overcome the high level of heterozygosity of the *P. fucata* genome.^15^ These initial genome reconstructions were useful in resolving biological questions such as the population structure of the species based on genome-wide SNPs.^26^ However, to accurately understand the complexity of the pearl oyster genome and for aquaculture improvement, e.g., genomic selection and GWAS, a more contiguous, high-quality genome resource with haplotype information is required.

Here we report a fully phased assembly of the *P. fucata* genome that was constructed using PacBio single-molecule real-time (SMRT) HiFi long reads and Omni-C chromosomal capture data. In addition, we also produced haplotype-merged genome assembly of *P. fucata* using a combination of sequencing technologies. We show that the quality and accuracy of this haplotype-phased assembly surpasses that of a haplotype-merged assembly, demonstrating that our haplotype-phased genome assembly strategy is effective in producing a nearly complete reference genome. Furthermore, the pearl oyster genome revealed unexpected diversity between haplotypes, especially in non-syntenic regions that may have a vital role in maintaining pearl oyster innate immunity.

## 2. Materials and methods

### 2.1. Materials

For haplotype-phased genome assembly, a wild individual kept in an aquaculture farm at Ainoshima Island, Fukuoka, Japan (hereafter we refer this individual as to AI) was used. For the haplotype-merged genome assembly, we obtained an inbred individual produced through three successive sib-mating generations at the Pearl Research Institute of K. MIKIMOTO & CO., LTD, Shima, Japan (MK). The experimental procedure and methods for the haplotype-merged MK assembly construction are described in Supplementary Text.

For specimen AI, an adductor muscle was sampled, immediately frozen in liquid nitrogen, and stored at −80°C until DNA extraction. High-molecular-weight genomic DNA was extracted using a Bionano Prep Blood and Cell Culture DNA isolation Kit (Bionano Genomics, CA, USA) following the manufacturer’s instructions. The size distribution and concentration of the extracted DNA were assessed using a FEMTO Pulse (Agilent Technologies, CA, USA) and a Qubit Fluorometer (Thermo Fisher Scientific, MA, USA) devices.

### 2.2. HiFi library construction, sequencing, and contig construction for the haplotype-phased assembly

Genomic DNA of AI was fragmented to a target size of 22 kb using a Megaruptor3 (Diagenode, Belgium). Fragmented DNA was then purified using AMPure PB (Pacific Biosciences, CA, USA). DNA fragment sizes were confirmed using a Femto Pulse System (Agilent Technologies). HiFi SMRTbell libraries were constructed using a SMRTbell Express Template Prep Kit 2.0 following the manufacturer’s protocol. Single-molecule sequencing was then conducted in circular consensus sequence (CCS) mode on a PacBio Sequel II platform. The quality of the obtained HiFi reads was confirmed based on *k*-mer (*k* = 40) spectra analysis with GeneScope.FK (https://github.com/thegenemyers/GENESCOPE.FK) and PloidyPlot (https://github.com/thegenemyers/MERQURY.FK), which are reimplemented and improved versions of GenomeScope 2.0 and SmudgePlot,^27^ respectively. We confirmed that both HiFi reads and Omni-C reads were generated from a single diploid genome (Supplementary Fig. S1C and D), with no contamination from other individuals or other species. HiFi reads were assembled using hifiasm version 0.13-r308^28^ with default parameters. The primary unitigs (*.p_utg.gfa), which contain nucleotide sequences of a pair of both haplotypes, generated by hifiasm were used as contigs that were subsequently scaffolded with Omni-C data.

### 2.3. Omni-C library preparation, sequencing, and scaffolding for final assembly

To construct a chromosome-scale assembly, we obtained long-range linkage information based on an *in vivo* chromatin conformation capture library produced with Dovetail’s endonuclease-based, proximity-ligation method called Omni-C. We further contracted them to perform data generation. The library was sequenced on an Illumina HiSeqX platform to approximately 30x sequence coverage. The quality of the Omni-C read set was confirmed with GeneScope.FK and PloidyPlot (*k* = 21) similarly to the HiFi reads.

Omni-C library sequences were aligned to the hifiasm assembly using bwa (https://github.com/lh3/bwa). Scaffolding was then performed by Dovetail using their HiRise pipeline.^29^ Distances between Omni-C read pairs mapped within contigs were analyzed with HiRise to produce a likelihood model for genomic distance between read pairs, and then this model was used to identify and break putative misjoins, to score prospective joins, and to make joins above a given threshold. Given the HiRise scaffolds, we then eliminated any remaining observable structural errors via manual inspection of the Omni-C contact map using Juicebox Assembly Tools.^30^

### 2.3. Quality assessment of the genome assembly

We basically followed criteria proposed in the Vertebrate Genome Project^31^ for quality assessment of the assembled scaffolds. We ran Merqury (v1.3)^32^ on the HiFi reads to calculate the *k*-mer (*k* = 20)-based quality value (QV). To estimate structural accuracy, we calculated the reliable block N50 length using Asset (v1.0.3; https://github.com/dfguan/asset). Mappings of HiFi reads and Omni-C reads for Asset were generated with Winnowmap^33^ and bwa (v0.7.17),^34^ respectively, using default parameters. To assess genome completeness, we ran BUSCO version 5.2.2^35^ with the metazoan_odb10 protein set (24 February 2021), using ‘--augustus’ option to predict gene sequences.

### 2.4. Repeat analysis

To develop a *de novo* repeat library, we ran RepeatModeler (version 2.0.1),^36^ which uses *ab initio* repeat prediction programs (RepeatScout 1.0.6^37^ and RECON 1.08^38^). Long terminal repeat (LTR) elements were identified using LTRharvest^39^ and LTR_retriever 2.9.0.^40^ These repeats were classified based on BLAST hits to the Repbase library (ver. 20181026) (https://www.girinst.org/)^41^ using RepeatMasker ver. 4.1.0 (http://www.repeatmasker.org/). Transposable elements (TEs) not classified by RepeatModeler were analyzed using DeepTE.^42^ The Kimura substitution rates of TEs were calculated using the Perl script calcDivergenceFromAlign.pl bundled in RepeatMasker. Tandem repeats were searched using Tandem Repeat Finder (TRF, ver. 4.09),^43^ and classified using the Tandem Repeats Analysis Program (TRAP, ver. 1.1.0).^44^

### 2.5. RNA-seq-guided and *de novo* gene predictions

Total RNA was isolated from frozen specimens using TRIzol reagent (Life Technologies, Carlsbad, CA, USA) according to the manufacturer’s protocol. Quality and quantity of RNA were checked using a Bioanalyzer (Agilent Technologies) and quantified using a Qubit fluorometer (Life Technologies, Waltham, MA, USA).

Strand-specific cDNA libraries of 15 developmental stages from unfertilized egg to umbo larva with eye spots (Supplementary Table S4) were constructed with TruSeq Stranded mRNA Library Prep (Illumina) reagents and sequenced on an Illumina HiSeq 4000 instrument. Raw sequence data were cleaned and quality-trimmed using Trimmomatic version 0.36^45^ with options ‘SLIDINGWINDOW:3:20 LEADING:3 TRAILING:3 HEADCROP:4 MINLEN:36’. In addition, RNA-seq data obtained from previous studies^15,46^ were also used for gene prediction. RNA-seq reads were mapped to the genome assembly using HiSAT2 (ver. 2.1.0)^47^ to generate a SAM file. The SAM file was sorted and transformed to a BAM file using samtools (ver. 1.6)^48^ and converted to GTF format using StringTie (ver. 2.1.5).^49^ Then, the GTF file and genome assembly were used as inputs for TransDecoder (ver. 5.5.0)^50^ to predict protein-coding regions in the genome. Quality-trimmed RNA-seq reads were assembled using Trinity (ver. 2.11.0).^51^

For Iso-Seq, total RNA was extracted from adult tissues and embryos at five different stages (Supplementary Table S5). Double-stranded cDNA was constructed using a NEBNext Single Cell/Low Input cDNA Synthesis & Amplification Module (New England Biolabs, USA), according to PacBio instructions. Size selection of the PCR product was performed using ProNex Size-Selective Chemistry (Promega Corporation, USA), and fragments of 2–8 kb were retained. Each SMRTbell library was constructed using the Pacific Biosciences SMRTbell Express Template Prep Kit 2.0. Fragment size distribution was confirmed on a Bioanalyzer High Sensitivity DNA Chip (Agilent Technologies) and quantified on a Qubit Fluorometer (Thermo Fisher Scientific). Iso-Seq sequencing was carried out on a PacBio Sequel II instrument using a Sequel II Sequencing 2.0 Kit and a SMRT Cell 8M Tray. Iso-Seq data processing was performed using the IsoSeq 3.1 software pipeline (Pacific Biosciences). CCSs were generated from subreads. Full-length non-concatemer reads were selected and clustered to obtain full-length isoforms. Polished isoforms were used in the following downstream analysis.

The transcriptome constructed from RNA-seq short reads and Iso-Seq reads was fed into the PASA (ver. 2.5) pipeline^52^ to build datasets for training and testing for gene prediction using Augustus (ver. 3.3.3).^53^ To generate hint files for Augustus, full-length cDNA sequences deposited at NCBI were Blast-searched against the genome assembly, and RNA-seq reads were mapped to the genome using STAR (ver. 2.7.8a).^54^ The genome assembly, with tandem repeats masked, was used for gene prediction using Augustus. Gene models were then analyzed using the InterProScan (ver. 5.14-53.0) platform^55^ to identify functional domains. Over-representing Pfam domains in non-syntenic regions of scaffold 9 (see section 3.4.) were identified using a hypergeometric test (*q*-value < 0.001), adjusted using the Benjamini–Hochberg method in R. Protein sequences were also Blastp-searched against both the nr and UniProt databases. We also ran BUSCO^56^ to assess the completeness of the gene models. Conserved sequence motifs were visualized using ggseqlogo.^57^

### 2.6. Conserved synteny between haplotypes

To identify syntenic blocks between 2 haplotypes, we ran MCScanX^58^ using Blastp results and gene arrangements with the following parameters: match_score: 50, match_size: 10, gap_penalty: −1, overlap_window: 5, E_value: 1e-05, max gaps: 25. *K*_a_ and *K*_s_ values of syntenic alleles were calculated using the add_kaks_to_MCScanX.pl script provided in the collinearity package (https://github.com/reubwn/collinearity).

### 2.7. Active autonomous transposons

Iso-seq sequences were mapped onto the haploid genome assemblies without repeat masking using pbmm2 (ver. 1.4.0, code at https://github.com/PacificBiosciences/pbmm2). Coding regions confirmed with the Iso-Seq reads were blastn-searched against the de novo TE database generated using RepeatModeler. Annotated genes were translated using TransDecoder (ver. 5.5.0)^50^ and protein sequences with more than 99 amino acids were analyzed using InterProScan (ver. 5.14-53.0)^55^ to identify functional domains. We examined genes having functional domains related to reverse transcriptase (RT), PIF1-like helicase, integrase, endonuclease, and recombinase.

### 2.8. Identification of genomic structural variations

PacBio HiFi reads were mapped using pbmm2 (ver. 1.4.0). Structural variants were called using the Pacific Biosciences caller PBSV (version 2.6.0, code at https://github.com/PacificBiosciences/pbsv), with default settings except for adding the ‘--tandem-repeats’ option in the ‘pbsv discover’ process to apply tandem repeat annotation generated from the TRAP results. Inserted sequences were blastn-searched against the *de novo* transposon database constructed from the *P. fucata* genome assembly using RepeatModeler.

## 3. Results

### 3.1. Genome assembly, quality assessment, and gene annotation

To establish a fully phased genome assembly of *P. fucata*, we sequenced genomic DNA of a wild individual from Ainoshima Island, Fukuoka, Japan (AI). We knew that the genome has significant sequence diversity between homologous chromosomes and expected that this high rate of heterozygosity (~3% according to GenomeScope) compared with the average error rate of PacBio SMRT HiFi sequencing reads (~0.2%) would allow us to assemble the two haplotypes separately. We also sequenced the genome of an inbred individual produced through three successive sib-mating generations cultured at the Pearl Research Institute of K. MIKIMOTO & CO., LTD, Shima, Japan (MK individual) to produce a haplotype-merged assembly for comparison (see Supplementary Text in detail for the MK assembly).

**Figure 1.**
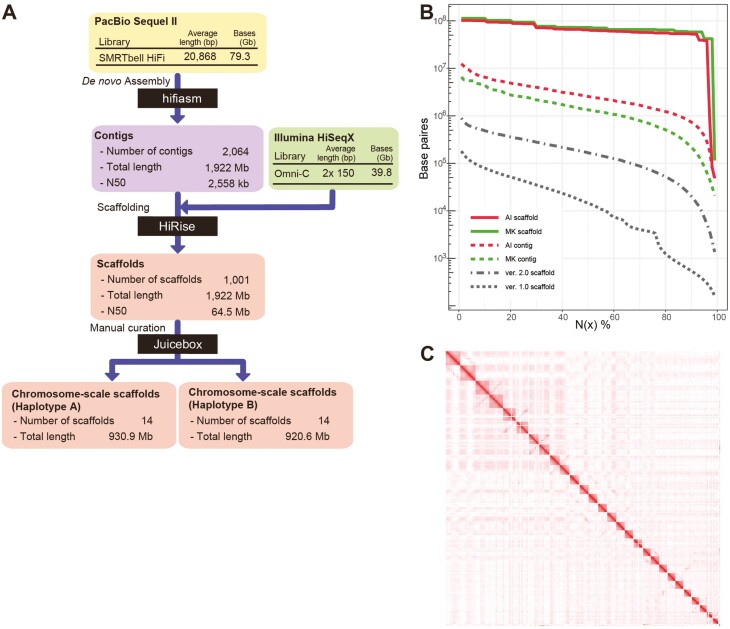
A haplotype-phased genome assembly of *Pinctada fucata*. (A) The sequencing and assembly pipeline to produce the haplotype-phased AI genome assembly. (B) An N(x) plot of four *P. fucata* genome assembly versions shows their relative contiguity. Versions 1.0 and 2.0 correspond to those reported in Takeuchi *et al*. (2012) and Takeuchi *et al.* (2016), respectively. The AI (haplotype-phased) and MK (haplotype-merged) assemblies are reported in this study. (C) A whole genome contact map showing 28 clusters representing chromosomal scaffolds. The colour scale is based on the relative interaction value from highest (1, red) to lowest value (<0.001, white).

From the AI genome, we obtained 3,799,879 HiFi long reads with an average length of 20.8 kbp (Fig. 1A, Supplementary Table S1). Altogether the HiFi reads constitute approximately 67.5× coverage of the *P. fucata* genome, the size of which is approximately 1.15 Gbp as measured using flow cytometry.^25^ An initial AI assembly, produced with hifiasm,^28^ consisted of 2,064 contigs with an N50 length of 2.56 Mb, showing substantial improvement over our previous *P. fucata* assemblies^15,25^ (Fig. 1B). Using an *in vivo* proximity-ligation method, Omni-C, we contracted the production of 34.6× coverage of the genome in 150-bp Illumina read pairs giving long-range linkage. These data were used to scaffold contigs with HiRise^29^ into 1,001 scaffolds with an N50 of 64.5 Mbp and an N90 of 52.3 Mbp. Finally, we manually curated the assembly based on contact map information at nine locations (Supplementary Fig. S2). The 28 largest scaffolds were deemed to constitute the final diploid chromosome set, leaving 71 Mbp (3.7%) of unassembled sequence in small scaffolds, likely filling the 1,160 gaps in the chromosome-designated scaffolds (Fig. 1C, Supplementary Table S2). We assessed the reads by PloidyPlot (https://github.com/thegenemyers/MERQURY.FK) and confirmed that both HiFi reads and Omni-C reads were indeed generated from a single diploid genome (Supplementary Fig. S1C and D), showing that there is no contamination from other individuals or other species. Analysis with Merqury^32^ using HiFi reads showed that the *k*-mer-based QV of the assembly is 65.0, i.e. 99.99997% accurate, and *k*-mer completeness reached 98.1%, outperforming any genome assemblies reported in the Vertebrate Genome Project (*k*-mer QV 33.6–44.5, *k*-mer completeness 87.2–98.1%).^31^ The N50 length of reliable blocks, in which no structural errors are thought to exist, was estimated with the Asset pipeline as 2.08 Mbp. These numbers demonstrate the high quality of the diploid assembly (Supplementary Table S2).

**Figure 2.**
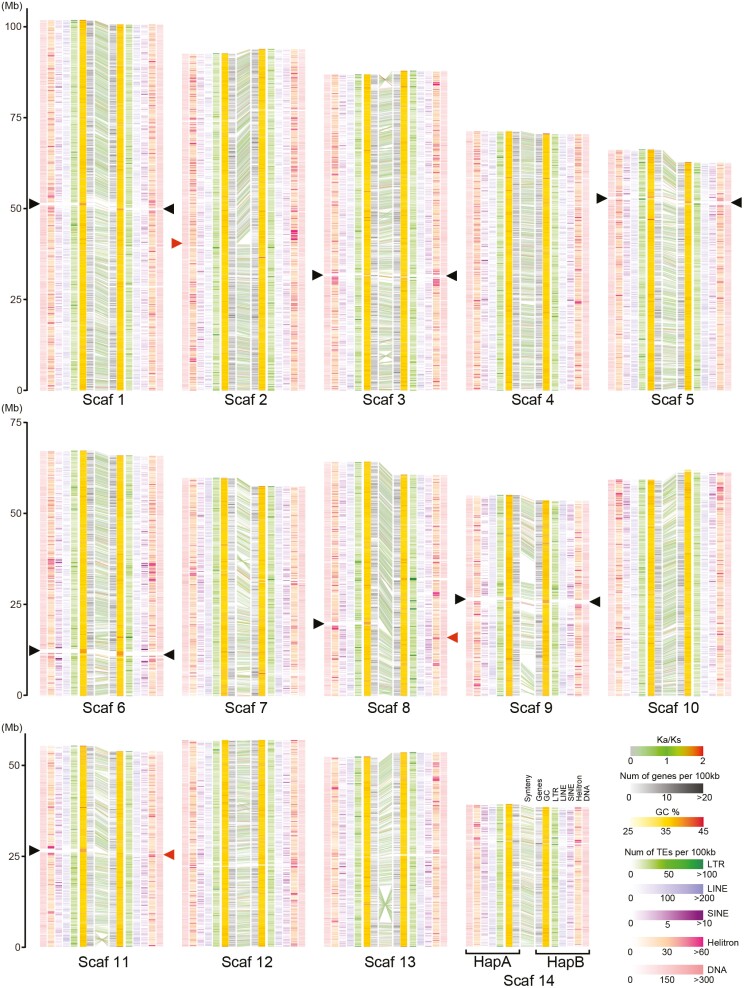
Collinearity between the 14 chromosomal scaffold pairs of haplotype A and B characterized by syntenic gene arrangement, gene density, GC%, and density of transposable elements including LTR, LINE, SINE, Helitron, and DNA transposons. Black arrowheads indicate putative centromere regions characterized by high GC% and the absence of protein-coding genes. Red arrowheads indicate examples of gap positions where repetitive sequences were missing in the scaffolds. See also supplementary Fig. S4.

The remainder of the analysis in this article focuses on the designated 28 chromosomes of the final AI assembly, all of which are longer than 38 Mbp. We conducted pairwise alignment of these scaffolds using MUMmer^59^ and identified 14 pairs of chromosomal scaffolds, which were then arbitrarily assigned to one of the chromosome-scale assemblies haplotype A or B in the order in which they appeared in the assembler software output file (Supplementary Fig. S3). The length difference of chromosomal scaffolds between haplotypes varies from 10 kb (scaffold 12) to 3.58 Mb (scaffold 8), averaging 1.6 Mb (Supplementary Table S3). The length difference between haplotypes is possibly due to sequence gaps in the scaffolds as well as structural variations (SVs) (Supplementary Fig. S4). Integrity of the two haplotype assemblies was assessed using Benchmarking Universal Single-Copy Orthologs (BUSCO) analysis with the metazoan dataset, showing that each haplotypes contained 95.2% and 95.9% of complete and single-copy BUSCOs (Supplementary Fig. S6), which are slightly higher than that of the haplotype-merged MK assembly (95.1%, Supplementary Table S2). The haplotype assemblies include 0.4% and 0.3% of complete and duplicated BUSCOs, which are lower than that of the MK assembly (1.0%). Both haploid genome assemblies showed the highest complete single-copy BUSCO and lowest complete duplicated BUSCO rate among published bivalve genomes (Supplementary Fig. S6). Based on the order of homologous genes, highly conserved synteny between haplotype pairs was confirmed (Fig. 2). These findings strongly imply that the 14 pairs of designated chromosome scaffolds, produced using HiFi long reads and Omni-C scaffolding, constitute a high-quality, nearly complete, haplotype-phased reconstruction of the genome of the AI pearl oyster.

Then, genemodel predictions were done for each haplotype. To this end, we obtained different types of genetic evidence, including RNA-seq short-read (Supplementary Tables S4) and Iso-Seq long-read transcriptomic data (Supplementary Tables S5) from various developmental stages and different tissues. We annotated 32,938 and 32,759 protein-coding genes for haplotypes A and B, respectively (Supplementary Table S6). BUSCO analysis demonstrated that assembled haplotypes A and B contain 94.6% and 96.2% complete metazoan BUSCO genes, respectively. These results showed that the assembly and annotation both comprise highly complete protein-coding sequences.

Based on the order of homologous genes, highly conserved synteny between the haplotype pairs was confirmed (Fig. 2). In addition, putative centromere regions were characterized in chromosomal scaffolds 1, 3, 5, 6, 9, and 11 by high GC% content and an absence of protein-coding genes. Megabase-scale inversions are also evident in chromosomal scaffolds 3, 7, 11, and 13 (Fig. 2 and Supplementary Fig. S3). Notably, scaffold 9 includes regions that lack synteny (Fig. 2) and pairwise alignment (Supplementary Figs S3I and S12). We confirmed that the assembly of these regions was correct because (i) there are few gaps in these regions and (ii) coverage of contact map information is comparable to that of other regions (Supplementary Figs S3 and S4).

In summary, the present sequencing and assembly strategy succeeded in constructing a fully phased, chromosome-scale genome assembly. To our knowledge, this *P. fucata* genome assembly outperforms previous pearl oyster genome assemblies^15,25,60^ and is one of the most accurate and continuous animal genome assemblies of any species.

### 3.2. Transposable elements

**Figure 3.**
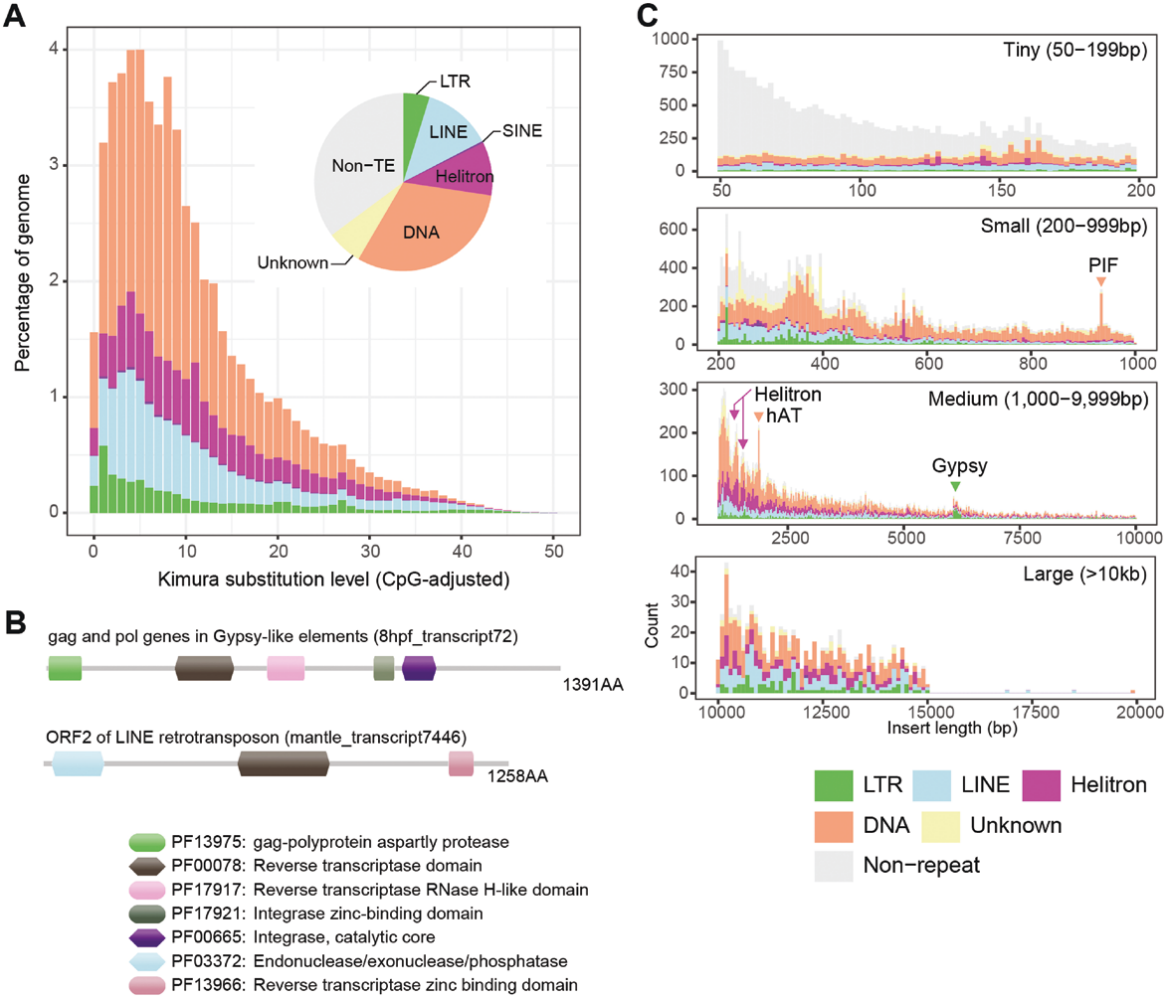
The recent expansion of transposable elements (TEs) shapes the pearl oyster genome. (A) A pie chart summarizes the TE content of the *P. fucata* genome assembly. A histogram shows the distribution of Kimura substitution levels among the TEs. The Kimura substitution level (%) for each copy compared with its consensus sequence is used as a proxy for the expansion history of the TEs. In general, TEs show low substitution levels, indicating their recent expansion. (B) Examples of domain architectures of TE-related gene products found in the Iso-Seq data. (C) The size distribution of insertions in haplotype A. TE copy insertions of similar size indicate that they are less degraded under neutral selection and that they have been inserted recently.

TEs are major components of the *P. fucata* genome. We confirmed that TEs constitute 53.3% of all nucleotides of the AI genome (Fig. 3A, Supplementary Figs S7 and S8, and Supplementary Table S7), which greatly surpasses the ver.1 assembly (1.8%), indicating that accurate long reads successfully reconstructed far more TEs. The most abundant retrotransposon subclass was LINE (11.58% of the total assembly), mainly composed of Penelope (10.34% of the total assembly). Among the LTR-retrotransposons, Gypsy (2.69% of the total assembly) was the most abundant in the *P. fucata* genome, consistent with a previous study.^61^ The other major element includes rolling-circle Helitron (8.42%), DNA transposons TcMar (6.49%), and hAT (5.26%). TEs are distributed unevenly in the *P. fucata* genome (Fig. 2). For example, Helitrons are often found in centromeric regions that typically show high GC contents and lack protein-coding genes. This tendency was reported in plant genomes.^62,63^

To examine evolution of the TEs in the *P. fucata* genome, we estimated Kimura substitution levels by comparing TE copy and its consensus sequence (Fig. 3A, Supplementary Fig. S8). Assuming that the TEs are under neutral conditions, the older the TE family, the more mutations it contains. The landscape of Kimura substitution levels showed that most TE sequence divergence was low (4-5%), implying either that TEs expanded in number recently or are currently active in the *P. fucata* genome. We applied the same TE annotation pipeline to selected bivalve genomes and found that TE proportions vary among bivalve species (Supplementary Fig. S8C–G). DNA transposons are the most abundant repeat component in all bivalve species examined. LINE is the second most abundant element in *P. fucata* and *M. edulis* genomes, presumably due to a recent expansion of LINE, as suggested by low Kimura substitution levels (Supplementary Fig. S8B and D). In comparison to other bivalves, the *P. fucata* genome is rich in LTR elements with low substitution levels, peaking at 1% and comprising 0.6% of the total assembly (Supplementary Fig. S8B).

Iso-Seq was used to confirm expression of genes for autonomous TEs (Fig. 3B, Supplementary Table S8). Autonomous transposons have open-reading frames that encode proteins necessary for transposition.^64^ The RT domain (PF00078), which is typically found in autonomous retrotransposons, was detected in all transcriptomes from the 5 developmental stages and 6 adult tissue types that we examined (Supplementary Fig. S9, Supplementary Table S8). We searched full-length Iso-Seq sequences that encode conserved functional domains of RT for autonomous retrotransposon and transposase for autonomous DNA transposons. Typical domain architectures of autonomous transposon gene products are shown in Fig. 3B. Transcripts encoding the RT domain are abundant in both embryos and adult tissues (55–215 transcripts per library), except for 1-h post-fertilization embryos, which contained only 4 transcripts. This indicates that TE-related transcripts are rarely, if ever carried from maternal tissue to the egg, and autonomous TE-related genes are zygotically expressed in later developmental stages, possibly after the maternal to zygotic transition.

### 3.3. Structural variations (SVs)

Our haplotype-resolved genome allowed us to accurately identify SVs within the genome. SVs include genomic rearrangements such as deletions, insertions, inversions, duplications, and translocations of more than 50 bp.^65^ To identify SVs, PacBio HiFi reads were mapped to the haplotype A assembly. Relative to haplotype B, the haplotype A assembly included 75,012 insertions (76.1 Mb) and 72,531 deletions (92.5 Mb), corresponding to 8.18% and 9.94% of the haploid assembly, respectively (Supplementary Figs S10 and S11, Supplementary Table S9). SVs may contribute, at least in part, to the differences in scaffold length between haplotypes (Supplementary Table S10). The size distribution of insertions showed sharp peaks at particular lengths, and these insertions were attributable to specific TEs (Fig. 3C). Among the inserted sequences longer than 199 bases, 38,283 of 46,245 sequences (82.8%) align to TEs in our *P. fucata* database by a Blastn search. Peaks around 950, 1,200–1,500, 2,000, and 6,000 bp correspond to PIF, Helitron, hAT, and Gypsy transposons, respectively. Those inserted elements of similar size represent intact copies; therefore, they emerged recently in the *P. fucata* genome, assuming that sequence identity is reduced over time under neutral selection. These results indicate that active TEs contribute significantly to the structural diversity between haplotypes.

### 3.4. The presence of non-syntenic regions in chromosomal scaffold 9

**Figure 4.**
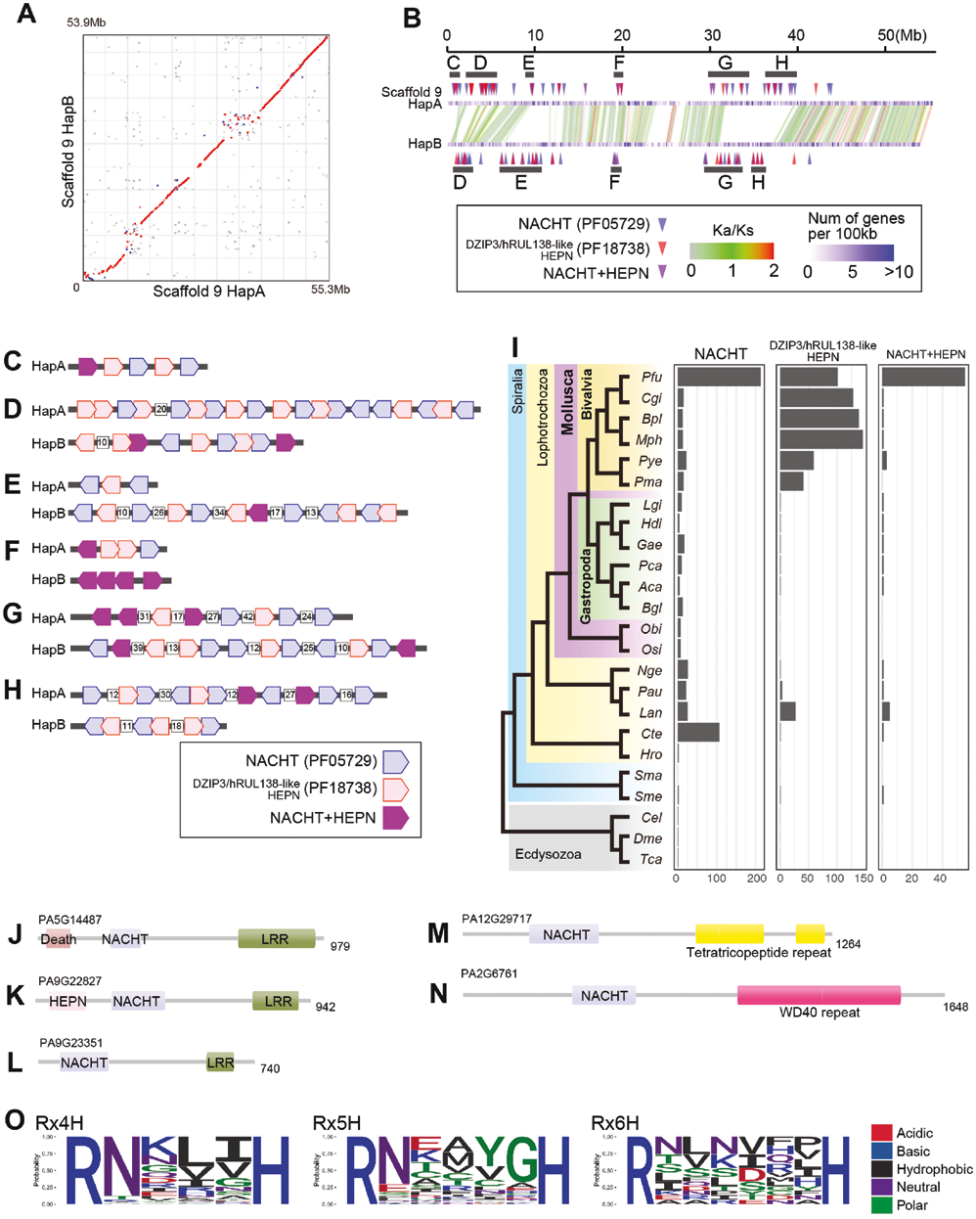
Non-syntenic regions in scaffold 9 contain an expanded repertoire of innate immunity genes. (A) Pairwise alignment of haplotypes A and B of scaffold 9. Aligned segments are represented as red (forward alignment) or blue (reverse alignment) dots. (B) Chromosomal positions of NACHT (PF05729) and DZIP3/hRUL138-like HEPN (PF18738) domain-containing protein (DCP) genes. Loci of NACHT DCP genes (red arrowheads), DZIP3/hRUL138-like HEPN DCP genes (blue arrowheads), and genes encoding both domains (purple arrowheads) are enriched in non-syntenic regions of scaffold 9. (C–H) The gene order of HEPN and DZIP3/hRUL138-like HEPN DCP genes is marked by grey lines in panel (B). Gene numbers are shown in white boxes if more than 9 genes intercalate in the tandem array of NACHT and DZIP3/hRUL138-like HEPN DCP genes. (I) Approximate numbers of NACHT and DZIP3/hRUL138-like DCP genes in Protostome species. In contrast to Ecdysozoa, there is wide copy gene number variation in Lophotorochozoa, ranging from 0 (*Schistosoma mansoni*) to 218 (*P. fucata*). The lineage-specific expansion of NACHT DCP genes in *P. fucata* is evident because the copy number is typically 5–20 in other molluscan species. Abbreviations of species names: Pfu, *Pinctada fucata*; Cgi, *Crassostrea gigas*; Bpl, *Bathymodiolus platifrons*; Mph, *Modiolus philippinarum*; Mye, *Mizuhopecten yessoensis*; Pma, *Pecten maximus*; Lgi, *Lottia gigantea*; Hdi, *Haliotis discus*; Gae, *Gigantopelta aegis*; Pca, *Pomacea canaliculata*; Aca, *Aplysia californica*; Bgl, *Biomphalaria glabrata*; Obi, *Octopus bimaculoides*; Osi, *Octopus sinensis*; Sph, *Sepia pharaonis*; Nge, *Notospermus geniculatus*; Pau, *Phoronis australis*; Lan, *Lingula anatina*; Cte, *Capitella teleta*; Hro, *Helobdella robusta*; Sma, *Schistosoma mansoni*; Sme, *Schmidtea mediterranea*; Cel, *Caenorhabditis elegans*; Dme, *Drosophila melanogaster*; Tca, *Tribolium castaneum*. (J–N) Examples of the diverse domain architectures of NACHT DCPs. The number of amino acids is shown at the right. (J, K) *P. fucata* NACHT DCPs with the typical tripartite domain architecture of NLRs, including a C-terminal ligand-sensing leucine-rich repeat (LRR) domain, a central nucleotide-binding NACHT domain, and an N-terminal effector domain. (O) Sequence logos for the consensus Rx4-6H motif in DZIP3/hRUL138-like HEPN domains. In the diploid *P. fucata* genome, 194 of 202 DZIP3/hRUL138-like HEPN DCPs include the typical Rx4-6H motif. Rx4H was the most common motif, found in 131 proteins. The presence of typical Rx4-6H motifs indicates RNase activity of the protein.

Sequence collinearity is conserved between haplotypes A and B over all chromosomes, as confirmed by *k*-mer-based sequence pairwise alignments (Supplementary Fig. S3) and synteny of protein-coding genes (Fig. 2). Exceptionally, chromosomal scaffold 9 has highly rearranged regions where no synteny was observed over 1 Mb between haplotypes (Figs 2 and 4A, Supplementary Figs S3I and S12). The total lengths of non-syntenic regions are 13.0 Mb in scaffold 9A and 15.4 Mb in scaffold 9B, occupying 23.5% and 28.6% of chromosomal scaffold 9, respectively. We analyzed enriched Pfam functional domains encoded in these regions (Supplementary Fig. S13, Supplementary Tables S11–17). Enriched Pfam domains include functional domains related to transposons, such as RT (PF00078), integrase (PF00665), Pao peptidase (PF05380), PIF1-like helicase (PF05970), and DDE endonuclease (PF13359). Haplotype-specific insertions of these mobile elements have disrupted the synteny between the haplotypes.

We also observed enrichment of immunity-related domains such as NACHT (NAIP, CIITA, HET-E, TP1),^66^ DZIP3/hRUL138-like HEPN (higher eukaryotes and prokaryotes nucleotide-binding domain),^67,68^ and immunoglobulin domains in non-syntenic regions (Fig. 4B, Supplementary Fig. S13). NACHT and DZIP3/hRUL138-like HEPN domain-containing protein (DCP) genes exhibit remarkable copy number variation and rearrangement between the two haplotypes (Fig. 4C–H). The *P. fucata* haploid genome contains more than 200 NACHT DCP genes, which is unusual among protostome genomes (Fig. 4I, Supplementary Table S18).

Gene expansion in the *P. fucata* genome allows diverse domain combinations with NACHT. In vertebrates, NACHT domains are found in nucleotide-binding domains, leucine-rich repeat (LRR)–containing receptors (NLRs),^69^ which are intracellular pattern recognition receptors to recognize various types of pathogens and damage-associated molecules.^70^ NLRs show a tripartite domain architecture of a C-terminal ligand-sensing LRR domain, a central nucleotide-binding NACHT domain, and an N-terminal effector domain, which is responsible for protein–protein interaction.^70^ A similar domain architecture is observed in the NACHT DCPs of *P. fucata*, often having a death domain or a DZIP3/hRUL138-like HEPN domain at the N-terminus (Fig. 4J and K). In particular, more than 50 NACHT DCPs possess DZIP3/hRUL138-like HEPN domains at their N-termini, which are overrepresented in the bivalve lineage (Fig. 4I and K, Supplementary Tables S18 and S19). In addition to LRRs, NACHT DCPs can carry repeat motifs, such as a tetratricopeptide repeat (TPR) or a WD40 repeat at their C-termini (Fig. 4M and N).

The HEPN superfamily contains conserved arginine and histidine residues separated by 4–6 variable amino acids (Rx4-6H), which is a putative RNase active site.^68^ In the diploid *P. fucata* genome, 194 of 202 DZIP3/hRUL138-like HEPN DCP include a typical Rx4-6H motif (Fig. 4O). Rx4H was the most common motif found in 131 proteins. The residue immediately after the conserved arginine was typically asparagine, as observed in diverse HEPN superfamily proteins.^68^

In the non-syntenic region near the centromere of chromosomal scaffold 9, immunoglobulin DCP genes are clustered (Supplementary Figs S14C and S15). Immunoglobulin DCPs often contain a fibronectin III domain and a transmembrane domain at their C-termini, while the number of immunoglobulin domains varies (Supplementary Fig. S15). We also analyzed synteny of expanded gene families related to innate immunity (C1q) and response to environmental stresses (heat shock protein 70, HSP70)^15^. C1q and HSP70 gene loci are consistent between haplotypes and have no significant presence/absence variation (Supplementary Fig. S14D and E).

### 3.5. Reduced heterozygosity by inbreeding

**Figure 5.**
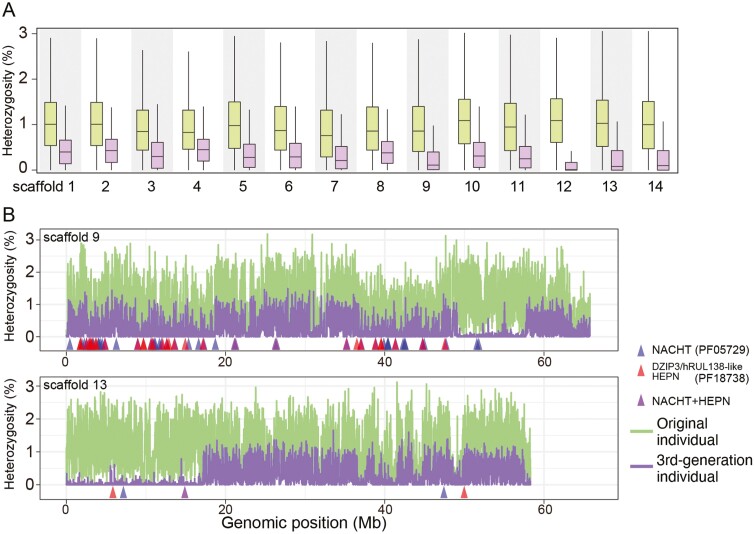
Reduced heterozygosity after successive inbreeding of the MK line. To calculate the heterozygosity rate, the number of SNPs per 10-kb, non-overlapping window was counted. (A) The heterozygosity rate in each chromosomal scaffold. Box plots for the original individual of inbreeding are shown in green, those of the third-generation individual in purple. (B) The extremely reduced heterozygosity in third-generation individual is exemplified by scaffolds 9 and 13. Megabase-scale ROH regions were observed from 49 to 58 Mb in scaffold 9 and from 0 to 18 Mb in scaffold 13, respectively, possibly due to autozygosity by inbreeding.

We observed a significant reduction of the heterozygosity rate after 3 consecutive full-sib matings in the MK strain (see Supplementary Text). *K*-mer-based estimates showed that the heterozygosity rate decreased from 3.57% to 2.55% (Supplementary Fig. S17). To count sites of single-nucleotide polymorphisms (SNPs), we mapped 60 Gbp of short reads (approximately 60 times the *P. fucata* haploid genome assembly size) from the original individual and a third-generation inbred individual to the MK assembly. After the 3 full-sib matings, the total number of SNP sites dropped from 10,141,582 to 3,634,878 (Fig. 5A). The SNP density was reduced throughout the chromosomes. Megabase-scale runs of homozygosity (ROH) were observed in chromosomal scaffolds 3, 6, 9, 10, 12, 13, and 14 (Fig. 5B, Supplementary Fig. S18).

## 4. Discussion

### 4.1. The haplotype-phased assembly strategy outperforms conventional haplotype-merged assembly

It is challenging to establish complete reference genome assemblies of bivalves due to their high heterozygosity and large numbers of repeat elements. Conventionally, genome assembly studies have endeavoured to remove redundancy in nucleotide sequences of highly heterozygotic diploid bivalve genomes to build more continuous genome assemblies, by using inbred individual Pacific oysters, *Crassostrea gigas*,^71^ and Manila clams, *Ruditapes philippinarum*,^18^ produced through sequential sister–brother matings or by using a hermaphroditic selfing scallop *Patinopecten yessoensis*.^72^ Afterward, advances in long-read sequencing technology have been applied to overcome the complexity of bivalve genomes. The major advantage of long-read sequencing is the ability to span low-complexity, repetitive regions. Furthermore, chromosome conformation capture technology can be used to cluster contigs using physical proximity information of the genome.^29^ The recent strategy for bivalve genome assembly is to combine multiple technologies, resulting in chromosome-scale assembly, which includes scaffolds with lengths of several tens of megabases, representing a large portion of total assembly size.^4,6,9,11,12,14^ In molluscs, gastropod genomes^3,5,7,10,13^ and a cephalopod species^8^ have also been assembled to chromosomal scale. Despite these chromosome-scale genome assembly efforts for molluscan species, all of them are haplotype-merged assembles; therefore, they did not fully investigate genetic variation in the respective species.

To establish a fully phased genome assembly, we sequenced genomic DNA from a wild individual, expecting that the genome would have significant sequence diversity between homologous chromosomes. The high rate of heterozygosity allows us to assemble separate haplotypes in the genome sequences. HiFi long-read sequences were assembled using the haplotype-resolved assembler hifiasm^28^ and proceeded to Omni-C scaffolding without a purging step, which is conventionally employed for genome assembly studies to eliminate one of the haplotypes. As a result, we succeeded in building a pair of chromosomal haploid genome sequences with hugely improved quality and contiguity compare to previous assemblies (Fig. 1B).

The quality of the haplotype-phased AI assembly is comparable to or even better than the haplotype-merged MK assembly (Fig. 1B), which was generated with a conventional sequencing strategy, using an inbred individual, combining different sequencing approaches, and purging duplicated sequences derived from the 2 haplotypes (Supplementary Text). For instance, complete and single-copy BUSCO scores of haplotype A (95.6%) and B (96.2%) assemblies are slightly higher than that of MK assembly (95.1%, Supplementary Table S2), indicating that each haploid assembly retains nearly complete information in terms of BUSCOs. Our haplotype-phasing approach using HiFi long-read and Omni-C scaffolding produced 2 sets of nearly complete reference genome assemblies. This method is straightforward and likely applicable to decode genomes of non-model organisms with high heterozygosity rates. Therefore, our haplotype-phasing approach provides an unprecedented opportunity to decode bivalve genomes, which are extremely challenging using conventional approaches.

### 4.2. Active transposon in the pearl oyster genome

TEs are major components of the *P. fucata* genome, comprising 53.3% of all nucleotides (Fig. 3A, Supplementary Fig. 7, and Supplementary Table S7). This result far exceeds the previous estimate of the ver.1 genome assembly, in which TEs comprised only 1.8%.^25^ This proportion was possibly underestimated because the previous assembly was constructed from medium to short reads generated using Roche 454 and Illumina GAIIx platforms, which do not span repetitive sequences.

Our Iso-Seq data indicate genes for autonomous TEs are expressed in all developmental stages and adult tissues examined (Fig. 3B, Supplementary Fig. S9, and Supplementary Table S8). LTR-retrotransposons usually encode 2 genes (*gag* and *pol*) in 1 or 2 open-reading frames.^73^ The Gag protein is involved in formation of virus-like particles. Pol protein contains functional domains responsible for transposition, including a protease, an integrase, a RT, and a RNaseH. LINE retrotransposons include ORF2, which encodes an endonuclease and an integrase.^74^ Expansion of copy numbers of RT genes and a low Kimura substitution level demonstrate that these TEs are currently active in the pearl oyster.

### 4.3. Various immune gene repertoires are retained in haplotypes

In general, a pair of homologous chromosomes is assumed to show collinearity of alleles across their length. This collinearity allows a physical exchange of alleles by recombination during meiosis, providing genomic diversity among siblings. The idea of collinearity between homologous chromosomes within a species has been challenged by studies of plant species.^75,76^ In non-syntenic regions, recombination cannot occur, resulting in reduced genome diversity in offspring. At the same time, non-syntenic regions can retain different gene repertoires in copy number and combination, resulting in haplotype variations in the population. Therefore, non-syntenic regions will consist of a balance between the cost of reduced recombination and the benefit of diversified gene repertoires.

The pearl oyster genome has regions lacking collinearity between haplotypes, which could not be recognized from conventional haplotype-merged genome assemblies of animals. Notably, the non-syntenic regions retain a remarkable gene repertoire of specific functional domains including NACHT, DZIP3/hRUL138-like HEPN, and Immunoglobulin domains (Fig. 4B–H, Supplementary Figs S13–15).

In vertebrates, NACHT domains are often associated with LRRs to constitute NLRs, which recognize intracellular pathogens to activate several innate immune pathways, including NF-κB signalling, IL-1β production, and apoptosis.^69,77^ In protostomes, NLRs are absent in fruit flies and nematodes,^69^ while the domain architecture is found in sea urchins^78^ and cnidarians.^79,80^ In the *P. fucata* genome, there are 19 genes encoding a central NACHT domain, an N-terminal death domain, and C-terminal LRRs (Fig. 4J, Supplementary Table S18). More than 50 NACHT DCPs possess a DZIP3/hRUL138-like HEPN domain at their N-termini, which is overrepresented in the bivalve lineage (Fig. 4I and K, Supplementary Tables S18 and S19). Based on their similarity to the tripartite domain architecture of canonical NLRs, these NACHT DCPs may be responsible for sensing pathogens within cells. NACHT DCP can also contain C-terminal repeat motifs, such as tetratricopeptide (TPR) and WD40 repeats. These domain architectures have been reported in cnidarian genomes,^80^ presumably a convergent event given their distant phylogenetic relationship. This shared domain architecture between distant lineages portends functional importance. Furthermore, NACHT DCPs represent variety of domain combinations, copy number variations, and presence/absence variations between haplotypes (Supplementary Tables S19 and S20). The NACHT domain is thought to interact with different types of NACHT DCPs, allowing a combinatorial variety of NACHT DCP oligomers with specificity for pathogens.^70,81^ The diverse gene repertoire in NACHT DCPs is possibly responsible for broad reactivity against pathogens, although its actual role needs to be examined by further analyses.

DZIP3/hRUL138-like HEPN DCPs without NACHT are also abundant in the pearl oyster genome (Fig. 4B–H). DZIP3/hRUL138-like HEPN domains contain a typical Rx4-6H motif (Fig. 4O), which is a putative RNase active site.^68^ It is possible that these DZIP3/hRUL138-like HEPN DCPs degrade RNAs of extraneous viruses and TEs. We also found copy number variation of Immunoglobulin (PF13927) genes between two haplotypes in one individual (Supplementary Figs. S14C and S15). This implies a large number of paralogues are maintained in the population. An immense gene repertoire can maximize the recognition capacities of diverse target sequences to countervail viral and microbial challenges.

In aquaculture, artificial selection can improve production efficiency by increasing survival and growth rates, and pearl quality, in the case of the pearl oysters. However, a major risk in artificial selection is inbreeding depression, which is caused by the increased homozygosity of deleterious recessive alleles or decreased heterozygosity at loci with overdominance, resulting in reduced fitness of offspring.^82^ Inbreeding depression in aquaculture has been reported in mollusc species such as *P. fucata*,^83^*Crassostrea gigas*,^84^*Ostrea edulis*,^85^*Tridacna squamosa*,^86^ and *Argopecten circularis*.^87^

We confirmed the reduced heterozygosity rate in the *P. fucata* genome after full-sib matings (Fig. 5, Supplementary Figs. S17 and S18). ROH in several scaffolds were evident, most likely due to autozygosity, in which parents transmit identical haplotypes from a common ancestor to their offspring.^88^ If autozygosity occurred in non-syntenic regions of the pearl oyster genome, it would cause reduced fitness of the individual by purging half of the innate immunity gene repertoire in these regions. It is notable that ROH was not observed in non-syntenic regions of scaffold 9 in the MK genome (Fig. 5B), indicating that this individual retains haplotype diversity in the non-syntenic region. Our haplotype-phased genome of the wild individual thus suggests that genetic variation in haplotypes is vital to maintaining innate immune capacity. We can test this hypothesis by analyzing a number of individual genomes to see whether there is selection pressure to maintain haplotype diversity in this region. If this hypothesis is correct, then it is essential to maintain genetic variation in the pearl oyster population for sustainable aquaculture.

We produced a fully phased, chromosome-scale genome assembly of *P. fucata* using PacBio HiFi long-read sequencing and Omni-C data. The present pearl oyster genome assembly is a substantial improvement over previous versions, providing one of the most contiguous and accurate animal genomes to date. Our haplotype-phased assembly strategy makes it possible to establish nearly complete reference genomes for non-model wild metazoans with high heterozygosity rates that are difficult to maintain in the laboratory. An assessment of the nearly complete haploid assembly revealed unexpected differences between its haplotypes: a considerable number of structural variants and megabase-scale non-syntenic regions. These non-syntenic regions contained remarkably diverse gene repertoires for innate pathogen detection, likely essential for survival. The presented pearl oyster genome assembly, therefore, provides pivotal information for understanding the complexity of the genome and the innate immunity of this economically important animal. Furthermore, these findings raise questions of whether haplotype diversity and the presence of non-syntenic regions are common to other animals, and what genomic evolutionary events generate this genomic complexity. More genome assemblies with haplotype phasing are essential to examine these questions.

## Supplementary Material

dsac035_suppl_Supplementary_Material

## Data Availability

All PacBio and Illumina reads are available from DRA accession numbers DRA013511, DRA013512, and DRA013527. Genome assemblies and predicted gene models are available at https://marinegenomics.oist.jp/gallery.

## References

[1] Romiguier, J., Gayral, P., Ballenghien, M., et al. 2014, Comparative population genomics in animals uncovers the determinants of genetic diversity, Nature, 515, 261–3. 25141177 10.1038/nature13685

[2] Ellegren, H., and Galtier, N. 2016, Determinants of genetic diversity, Nat. Rev. Genet., 17, 422–33. 27265362 10.1038/nrg.2016.58

[3] Liu, C., Zhang, Y., Ren, Y., et al. 2018, The genome of the golden apple snail *Pomacea canaliculata* provides insight into stress tolerance and invasive adaptation, GigaScience, 7, 1–13. 10.1093/gigascience/giy101 PMC6129957 30107526

[4] Bai, C.-M., Xin, L.-S., Rosani, U., et al. 2019, Chromosomal-level assembly of the blood clam, *Scapharca* (*Anadara*) *broughtonii*, using long sequence reads and Hi-C, GigaScience, 8, 1–8. 10.1093/gigascience/giz067 PMC6615981 31289832

[5] Guo, Y., Zhang, Y., Liu, Q., et al. 2019, A chromosomal-level genome assembly for the giant African snail *Achatina fulica*, GigaScience, 8, 1–8. 10.1093/gigascience/giz124 PMC6802634 31634388

[6] Ran, Z., Li, Z., Yan, X., et al. 2019, Chromosome-level genome assembly of the razor clam *Sinonovacula constricta* (Lamarck, 1818), Mol. Ecol. Resour., 19, 1647–58. 31483923 10.1111/1755-0998.13086

[7] Sun, J., Chen, C., Miyamoto, N., et al. 2020, The Scaly-foot Snail genome and implications for the origins of biomineralised armour, Nat. Commun., 11, 1–12. 32269225 10.1038/s41467-020-15522-3 PMC7142155

[8] Li, F., Bian, L., Ge, J., et al. 2020, Chromosome-level genome assembly of the East Asian common octopus (*Octopus sinensis*) using PacBio sequencing and Hi-C technology, Mol. Ecol. Resour., 20, 1572–82. 32603549 10.1111/1755-0998.13216

[9] Ip, J.C., Xu, T., Sun, J., et al. 2021, Host–endosymbiont genome integration in a deep-sea chemosymbiotic clam, Mol. Biol. Evol., 38, 502–18. 32956455 10.1093/molbev/msaa241 PMC7826175

[10] Lan, Y., Sun, J., Chen, C., et al. 2021, Hologenome analysis reveals dual symbiosis in the deep-sea hydrothermal vent snail *Gigantopelta aegis*, Nat. Commun., 12, 1165. 33608555 10.1038/s41467-021-21450-7 PMC7895826

[11] Peñaloza, C., Gutierrez, A.P., Eöry, L., et al. 2021, A chromosome-level genome assembly for the Pacific oyster *Crassostrea gigas*, GigaScience, 10, 1–9. 10.1093/gigascience/giab020 PMC7992393 33764468

[12] Teng, W., Xie, X., Nie, H., et al. 2021, Chromosome-level genome assembly of *Scapharca kagoshimensis* reveals the expanded molecular basis of heme biosynthesis in ark shells, Mol. Ecol. Resour., 00, 1–12. 10.1111/1755-0998.13460 34214251

[13] Liu, C., Ren, Y., Li, Z., et al. 2021, Giant African snail genomes provide insights into molluscan whole-genome duplication and aquatic–terrestrial transition, Mol. Ecol. Resour., 21, 478–94. 33000522 10.1111/1755-0998.13261

[14] Yang, J.-L., Feng, D.-D., Liu, J., et al. 2021, Chromosome-level genome assembly of the hard-shelled mussel *Mytilus coruscus*, a widely distributed species from the temperate areas of East Asia, GigaScience, 10, giab024. 33891010 10.1093/gigascience/giab024 PMC8063583

[15] Takeuchi, T., Koyanagi, R., Gyoja, F., et al. 2016, Bivalve-specific gene expansion in the pearl oyster genome: implications of adaptation to a sessile lifestyle, Zool. Lett., 2, 3. 10.1186/s40851-016-0039-2 PMC4759782 26900483

[16] Sun, J., Zhang, Y., Xu, T., et al. 2017, Adaptation to deep-sea chemosynthetic environments as revealed by mussel genomes, Nat. Ecol. Evol., 1, 0121. 10.1038/s41559-017-0121 28812709

[17] Powell, D., Subramanian, S., Suwansa-ard, S., et al. 2018, The genome of the oyster *Saccostrea* offers insight into the environmental resilience of bivalves, DNA Res., 25, 655–65. 30295708 10.1093/dnares/dsy032 PMC6289776

[18] Yan, X., Nie, H., Huo, Z., et al. 2019, Clam genome sequence clarifies the molecular basis of its benthic adaptation and extraordinary shell color diversity, iScience, 19, 1225–37. 31574780 10.1016/j.isci.2019.08.049 PMC6831834

[19] Gerdol, M., Moreira, R., Cruz, F., et al. 2020, Massive gene presence–absence variation shapes an open pan-genome in the Mediterranean mussel, Genome Biol., 21, 275. 33168033 10.1186/s13059-020-02180-3 PMC7653742

[20] Nagai, K. 2013, A history of the cultured pearl industry, JZOO, 30, 783–93. 10.2108/zsj.30.783 24125642

[21] Morizane, T., Takimoto, S., Nishikawa, S., et al. 2001, Mass mortalities of Japanese Pearl Oyster in Uwa Sea, Ehime in 1997–1999, Fish Pathol., 36, 207–16.

[22] Matsuyama, T., Yasuike, M., Fujiwara, A., et al. 2017, A spirochaete is suggested as the causative agent of Akoya oyster disease by metagenomic analysis, PLoS One, 12, e0182280. 28771537 10.1371/journal.pone.0182280 PMC5542438

[23] Matsuyama, T., Miwa, S., Mekata, T., et al. 2021, Mass mortality of pearl oyster (*Pinctada fucata* (Gould)) in Japan in 2019 and 2020 is caused by an unidentified infectious agent, PeerJ, 9, e12180. 34616626 10.7717/peerj.12180 PMC8462378

[24] Sakatoku, A., Hatano, K., Tanaka, S. and Isshiki, T. 2021, Isolation and characterization of a *Vibrio* sp. strain MA3 associated with mass mortalities of the pearl oyster *Pinctada fucata*, Arch. Microbiol., 203, 5267–73. 34216219 10.1007/s00203-021-02457-6

[25] Takeuchi, T., Kawashima, T., Koyanagi, R., et al. 2012, Draft genome of the pearl oyster *Pinctada fucata*: a platform for understanding bivalve biology, DNA Res., 19, 117–30. 22315334 10.1093/dnares/dss005 PMC3325083

[26] Takeuchi, T., Masaoka, T., Aoki, H., et al. 2020, Divergent northern and southern populations and demographic history of the pearl oyster in the western Pacific revealed with genomic SNPs, Evol. Appl., 13, 837–53. 32211071 10.1111/eva.12905 PMC7086055

[27] Ranallo-Benavidez, T.R., Jaron, K.S., Schatz, M.C. 2020, GenomeScope 2.0 and Smudgeplot for reference-free profiling of polyploid genomes, Nat. Commun., 11, 1432. 32188846 10.1038/s41467-020-14998-3 PMC7080791

[28] Cheng, H., Concepcion, G.T., Feng, X., Zhang, H., and Li, H. 2021, Haplotype-resolved de novo assembly using phased assembly graphs with hifiasm, Nat. Methods, 18, 170–5. 33526886 10.1038/s41592-020-01056-5 PMC7961889

[29] Putnam, N.H., O’Connell, B.L., Stites, J.C., et al. 2016, Chromosome-scale shotgun assembly using an in vitro method for long-range linkage, Genome Res., 26, 342–50. 26848124 10.1101/gr.193474.115 PMC4772016

[30] Durand, N.C., Robinson, J.T., Shamim, M.S., et al. 2016, Juicebox provides a visualization system for hi-c contact maps with unlimited zoom, Cell Syst., 3, 99–101. 27467250 10.1016/j.cels.2015.07.012 PMC5596920

[31] Rhie, A., McCarthy, S.A., Fedrigo, O., et al. 2021, Towards complete and error-free genome assemblies of all vertebrate species, Nature, 592, 737–46. 33911273 10.1038/s41586-021-03451-0 PMC8081667

[32] Rhie, A., Walenz, B.P., Koren, S., and Phillippy, A.M. 2020, Merqury: reference-free quality, completeness, and phasing assessment for genome assemblies, Genome Biol., 21, 245. 32928274 10.1186/s13059-020-02134-9 PMC7488777

[33] Jain, C., Rhie, A., Zhang, H., et al. 2020, Weighted minimizer sampling improves long read mapping, Bioinformatics, 36, i111–8. 32657365 10.1093/bioinformatics/btaa435 PMC7355284

[34] Li, H. and Durbin, R. 2009, Fast and accurate short read alignment with Burrows–Wheeler transform, Bioinformatics, 25, 1754–60. 19451168 10.1093/bioinformatics/btp324 PMC2705234

[35] Simão, F.A., Waterhouse, R.M., Ioannidis, P., Kriventseva, E.V. and Zdobnov, E.M. 2015, BUSCO: assessing genome assembly and annotation completeness with single-copy orthologs, Bioinformatics, 31, 3210–2. 26059717 10.1093/bioinformatics/btv351

[36] Flynn, J.M., Hubley, R., Goubert, C., et al. 2020, Repeatmodeler2 for automated genomic discovery of transposable element families, PNAS, 117, 9451–7. 32300014 10.1073/pnas.1921046117 PMC7196820

[37] Price, A.L., Jones, N.C. and Pevzner, P.A. 2005, De novo identification of repeat families in large genomes, Bioinformatics, 21, i351–8. 15961478 10.1093/bioinformatics/bti1018

[38] Bao, Z. and Eddy, S.R. 2002, Automated *de novo* identification of repeat sequence families in sequenced genomes, Genome Res., 12, 1269–76. 12176934 10.1101/gr.88502 PMC186642

[39] Ellinghaus, D., Kurtz, S. and Willhoeft, U. 2008, LTRharvest, an efficient and flexible software for de novo detection of LTR retrotransposons, BMC Bioinf., 9, 18. 10.1186/1471-2105-9-18 PMC2253517 18194517

[40] Ou, S. and Jiang, N. 2018, LTR_retriever: a highly accurate and sensitive program for identification of long terminal repeat retrotransposons, Plant Physiol., 176, 1410–22. 29233850 10.1104/pp.17.01310 PMC5813529

[41] Bao, W., Kojima, K.K. and Kohany, O. 2015, Repbase update, a database of repetitive elements in eukaryotic genomes, Mobile DNA, 6, 11. 26045719 10.1186/s13100-015-0041-9 PMC4455052

[42] Yan, H., Bombarely, A. and Li, S. 2020, DeepTE: a computational method for de novo classification of transposons with convolutional neural network, Bioinformatics, 36, 4269–75. 32415954 10.1093/bioinformatics/btaa519

[43] Benson, G. 1999, Tandem repeats finder: a program to analyze DNA sequences, Nucleic Acids Res., 27, 573–80. 9862982 10.1093/nar/27.2.573 PMC148217

[44] Sobreira, T.J.P., Durham, A.M. and Gruber, A. 2006, TRAP: automated classification, quantification and annotation of tandemly repeated sequences, Bioinformatics, 22, 361–2. 16332714 10.1093/bioinformatics/bti809

[45] Bolger, A.M., Lohse, M. and Usadel, B. 2014, Trimmomatic: a flexible trimmer for illumina sequence data, Bioinformatics, 30, 2114–20. 24695404 10.1093/bioinformatics/btu170 PMC4103590

[46] Zhao, R., Takeuchi, T., Luo, Y.-J., et al. 2018, Dual gene repertoires for larval and adult shells reveal molecules essential for molluscan shell formation, Mol. Biol. Evol., 35, 2751–61. 30169718 10.1093/molbev/msy172 PMC6231486

[47] Kim, D., Paggi, J.M., Park, C., Bennett, C. and Salzberg, S.L. 2019, Graph-based genome alignment and genotyping with HISAT2 and HISAT-genotype, Nat. Biotechnol., 37, 907–15. 31375807 10.1038/s41587-019-0201-4 PMC7605509

[48] Danecek, P., Bonfield, J.K., Liddle, J., et al. 2021, Twelve years of SAMtools and BCFtools, GigaScience, 10, 1–4. 10.1093/gigascience/giab008 PMC7931819 33590861

[49] Kovaka, S., Zimin, A.V., Pertea, G.M., et al. 2019, Transcriptome assembly from long-read RNA-seq alignments with StringTie2, Genome Biol., 20, 278. 31842956 10.1186/s13059-019-1910-1 PMC6912988

[50] Haas, B.J., Papanicolaou, A., Yassour, M., et al. 2013, De novo transcript sequence reconstruction from RNA-seq using the Trinity platform for reference generation and analysis, Nat. Protoc., 8, 1494–512. 23845962 10.1038/nprot.2013.084 PMC3875132

[51] Grabherr, M.G., Haas, B.J., Yassour, M., et al. 2011, Full-length transcriptome assembly from RNA-Seq data without a reference genome, Nat. Biotechnol., 29, 644–52. 21572440 10.1038/nbt.1883 PMC3571712

[52] Haas, B.J., Delcher, A.L., Mount, S.M., et al. 2003, Improving the *Arabidopsis* genome annotation using maximal transcript alignment assemblies, Nucleic Acids Res., 31, 5654–66. 14500829 10.1093/nar/gkg770 PMC206470

[53] Stanke, M., Schöffmann, O., Morgenstern, B. and Waack, S. 2006, Gene prediction in eukaryotes with a generalized hidden Markov model that uses hints from external sources, BMC Bioinf., 7, 62. 10.1186/1471-2105-7-62 PMC1409804 16469098

[54] Dobin, A., Davis, C.A., Schlesinger, F., et al. 2013, STAR: ultrafast universal RNA-seq aligner, Bioinformatics, 29, 15–21. 23104886 10.1093/bioinformatics/bts635 PMC3530905

[55] Jones, P., Binns, D., Chang, H.-Y., et al. 2014, InterProScan 5: genome-scale protein function classification, Bioinformatics, 30, 1236–40. 24451626 10.1093/bioinformatics/btu031 PMC3998142

[56] Manni, M., Berkeley, M.R., Seppey, M., Simão, F.A. and Zdobnov, E.M. 2021, BUSCO Update: novel and streamlined workflows along with broader and deeper phylogenetic coverage for scoring of eukaryotic, prokaryotic, and viral genomes, Mol. Biol. Evol., 38, 4647–54. 34320186 10.1093/molbev/msab199 PMC8476166

[57] Wagih, O. 2017, ggseqlogo: a versatile R package for drawing sequence logos, Bioinformatics, 33, 3645–7. 29036507 10.1093/bioinformatics/btx469

[58] Wang, Y., Tang, H., DeBarry, J.D., et al. 2012, MCScanX: a toolkit for detection and evolutionary analysis of gene synteny and collinearity, Nucleic Acids Res., 40, e49–e49. 22217600 10.1093/nar/gkr1293 PMC3326336

[59] Kurtz, S., Phillippy, A., Delcher, A. L., et al. 2004, Versatile and open software for comparing large genomes. Genome Biol., 5, R12. 14759262 10.1186/gb-2004-5-2-r12 PMC395750

[60] Du, X., Fan, G., Jiao, Y., et al. 2017, The pearl oyster *Pinctada fucata martensii* genome and multi-omic analyses provide insights into biomineralization, GigaScience, 6, 1–12. 10.1093/gigascience/gix059 PMC5597905 28873964

[61] Thomas-Bulle, C., Piednoël, M., Donnart, T., et al. 2018, Mollusc genomes reveal variability in patterns of LTR-retrotransposons dynamics, BMC Genomics, 19, 821. 30442098 10.1186/s12864-018-5200-1 PMC6238403

[62] Yang, L. and Bennetzen, J.L. 2009, Structure-based discovery and description of plant and animal Helitrons, PNAS, 106, 12832–7. 19622734 10.1073/pnas.0905563106 PMC2722332

[63] Hu, K., Xu, K., Wen, J., et al. 2019, Helitron distribution in Brassicaceae and whole Genome Helitron density as a character for distinguishing plant species, BMC Bioinf., 20, 354. 10.1186/s12859-019-2945-8 PMC6591975 31234777

[64] Wessler, S.R. 2006, Transposable elements and the evolution of eukaryotic genomes, PNAS, 103, 17600–1. 17101965 10.1073/pnas.0607612103 PMC1693792

[65] Sudmant, P.H., Rausch, T., Gardner, E.J., et al. 2015, An integrated map of structural variation in 2,504 human genomes, Nature, 526, 75–81. 26432246 10.1038/nature15394 PMC4617611

[66] Koonin, E.V. and Aravind, L. 2000, The NACHT family—a new group of predicted NTPases implicated in apoptosis and MHC transcription activation, Trends Biochem. Sci., 25, 223–4. 10782090 10.1016/s0968-0004(00)01577-2

[67] Grynberg, M., Erlandsen, H. and Godzik, A. 2003, HEPN: a common domain in bacterial drug resistance and human neurodegenerative proteins, Trends Biochem. Sci., 28, 224–6. 12765831 10.1016/S0968-0004(03)00060-4

[68] Anantharaman, V., Makarova, K.S., Burroughs, A.M., Koonin, E.V. and Aravind, L. 2013, Comprehensive analysis of the HEPN superfamily: identification of novel roles in intra-genomic conflicts, defense, pathogenesis and RNA processing, Biol. Direct, 8, 15. 23768067 10.1186/1745-6150-8-15 PMC3710099

[69] Jones, J.D.G., Vance, R.E. and Dangl, J.L. 2016, Intracellular innate immune surveillance devices in plants and animals, Science, 354, 1117. 10.1126/science.aaf6395 27934708

[70] Fritz, J.H., Ferrero, R.L., Philpott, D.J. and Girardin, S.E. 2006, Nod-like proteins in immunity, inflammation and disease, Nat. Immunol., 7, 1250–7. 17110941 10.1038/ni1412

[71] Zhang, G., Fang, X., Guo, X., et al. 2012, The oyster genome reveals stress adaptation and complexity of shell formation, Nature, 490, 49–54. 22992520 10.1038/nature11413

[72] Wang, S., Zhang, J., Jiao, W., et al. 2017, Scallop genome provides insights into evolution of bilaterian karyotype and development, Nat. Ecol. Evol., 1, 0120. 10.1038/s41559-017-0120 PMC10970998 28812685

[73] Gao, X., Havecker, E.R., Baranov, P.V., Atkins, J.F. and Voytas, D.F. 2003, Translational recoding signals between gag and pol in diverse LTR retrotransposons, RNA, 9, 1422–30. 14623998 10.1261/rna.5105503 PMC1370496

[74] Wicker, T., Sabot, F., Hua-Van, A., et al. 2007, A unified classification system for eukaryotic transposable elements, Nat. Rev. Genet., 8, 973–82. 17984973 10.1038/nrg2165

[75] de Boer, J.M., Datema, E., Tang, X., et al. 2015, Homologues of potato chromosome 5 show variable collinearity in the euchromatin, but dramatic absence of sequence similarity in the pericentromeric heterochromatin, BMC Genomics, 16, 374. 25958312 10.1186/s12864-015-1578-1 PMC4470070

[76] Jiao, W.-B. and Schneeberger, K. 2020, Chromosome-level assemblies of multiple Arabidopsis genomes reveal hotspots of rearrangements with altered evolutionary dynamics, Nat. Commun., 11, 989. 32080174 10.1038/s41467-020-14779-y PMC7033125

[77] Chen, G., Shaw, M.H., Kim, Y.-G. and Nuñez, G. 2009, NOD-like receptors: role in innate immunity and inflammatory disease, Ann. Rev. Pathol., 4, 365–98. 18928408 10.1146/annurev.pathol.4.110807.092239

[78] Hibino, T., Loza-Coll, M., Messier, C., et al. 2006, The immune gene repertoire encoded in the purple sea urchin genome, Dev. Biol., 300, 349–65. 17027739 10.1016/j.ydbio.2006.08.065

[79] Hamada, M., Shoguchi, E., Shinzato, C., et al. 2013, The complex NOD-like receptor repertoire of the coral *Acropora digitifera* includes novel domain combinations, Mol. Biol. Evol., 30, 167–76. 22936719 10.1093/molbev/mss213

[80] Emery, M.A., Dimos, B.A. and Mydlarz, L.D. 2021, Cnidarian pattern recognition receptor repertoires reflect both phylogeny and life history traits, Front. Immunol., 12, 2430. 10.3389/fimmu.2021.689463 PMC8260672 34248980

[81] Damiano, J.S., Oliveira, V., Welsh, K. and Reed, J.C. 2004, Heterotypic interactions among NACHT domains: implications for regulation of innate immune responses, Biochem. J., 381, 213–9. 15107016 10.1042/BJ20031506 PMC1133779

[82] Charlesworth, D. and Willis, J.H. 2009, The genetics of inbreeding depression, Nat. Rev. Genet., 10, 783–96. 19834483 10.1038/nrg2664

[83] Wada, K.T. and Komaru, A. 1994, Effect of selection for shell coloration on growth rate and mortality in the Japanese pearl oyster, *Pinctada fucata martensii*, Aquaculture, 125, 59–65.

[84] Evans, F., Matson, S., Brake, J. and Langdon, C. 2004, The effects of inbreeding on performance traits of adult Pacific oysters (*Crassostrea gigas*), Aquaculture, 230, 89–98.

[85] Naciri-Graven, Y., Launey, S., Lebayon, N., Gerard, A. and Baud, J.-P. 2000, Influence of parentage upon growth in *Ostrea edulis*: evidence for inbreeding depression, Genetics Res., 76, 159–68. 11132409 10.1017/s0016672300004663

[86] Zhang, Y., Ma, H., Li, X., et al. 2020, Analysis of inbreeding depression on performance traits of three giant clams (*Tridacna derasa*, *T. squamosa*, and *T. crocea*) in the South China Sea, Aquaculture, 521, 735023.

[87] Ibarra, A.M., Cruz, P. and Romero, B.A. 1995, Effects of inbreeding on growth and survival of self-fertilized catarina scallop larvae, *Argopecten circularis*, Aquaculture, 134, 37–47.

[88] Curik, I., Ferenčaković, M., and Sölkner, J. 2014, Inbreeding and runs of homozygosity: A possible solution to an old problem. Livestock Sci., 166, 26–34.

