# Supplementary material for: A high-quality, haplotype-phased genome reconstruction reveals unexpected haplotype diversity in a pearl oyster
Source: DNA Res. 2022 Nov 10;29(6):dsac035. doi: 10.1093/dnares/dsac035 (PMC9646362; doi:10.1093/dnares/dsac035)
Supplement: dsac035_suppl_Supplementary_Material [file dsac035_suppl_supplementary_material.pdf]

Supplemental Material for

A high-quality, haplotype-phased genome reconstruction reveals unexpected haplotype diversity in a pearl oyster

Takeshi Takeuchi[¶*], Yoshihiko Suzuki[¶], Shugo Watabe, Kiyohito Nagai, Tetsuji Masaoka, Manabu Fujie, Mayumi Kawamitsu, Noriyuki Satoh, and Eugene W. Myers

[¶] The first two authors contributed equally to this work.

*Corresponding author. Email: t.takeuchi@oist.jp

Contents

Supplementary Text: Haplotype-merged genome assembly construction

Supplementary Figs. S1 to S18

Supplementary Tables S1 to S20

References

Supplementary Note. Haplotype-merged genome assembly construction

1. DNA extraction

In preparation for genome sequencing to construct a haplotype-merged genome assembly, we used a *Pinctada fucata* strain inbred through several generations. Accordingly, we expected a reduction of heterozygosity. The inbred line originated from the individual used in our previous genome sequencing project[1,2]. The strain was cultured at the Pearl Research Institute of K. MIKIMOTO & CO., LTD (Shima, Japan). A third-generation inbred individual (hereafter we refer to this individual as MK) was used for genome sequencing. An adductor muscle was sampled and immediately frozen in liquid nitrogen, and kept at -80˚C until DNA extraction. High-molecular-weight genomic DNA was extracted using a Bionano Prep Blood and Cell Culture DNA isolation Kit (Bionano Genomics, CA, USA) following the manufacturer's instructions. The size distribution and concentration of the DNA were assessed using a Femto Pulse System (Agilent Technologies, CA, USA) and a Qubit Fluorometer (Thermo Fisher Scientific, MA, USA).

2. Long-read library preparation and sequencing

The genomic DNA of AI was fragmented to the target size of 30 kb using Megaruptor (Diagenode, Belgium). Fragmented DNA was purified using AMPure PB (Pacific Biosciences, CA, USA). DNA fragment sizes were estimated using Femto Pulse System (Agilent Technologies). SMRTbell libraries were constructed using a

SMRT Bell template prep kit 1.0 following the manufacturer’s protocol. The SMRTbell library was sequenced on a PacBio RSII instrument with P6 polymerase binding and C4 chemistry kits (P6C4).

3. Short-read library preparation and sequencing

A paired-end library was constructed using a NEBNext Ultra II FS DNA Library Prep Kit for Illumina (New England Biolabs) following the standard protocol provided by Illumina. For 10X Genomics library preparation, high-molecular-weight DNA was loaded into the 10X Chromium Controller. A 10X Genomics library was constructed using the Chromium Genome Reagent Kit v2 (10X Genomics, CA, USA), following the manufacturer's recommended protocol. These libraries were sequenced using the Illumina NovaSeq6000 SP platform. Raw reads were processed using Trimmomatic v0.36[3] in order to remove adaptor sequences and to trim low-quality bases with an average quality score lower than 20 in a sliding window of four bases.

In order to obtain long-range linkages using *in vivo* chromatin conformation capture library, a frozen adductor muscle, from the same specimen used for sequencing mentioned above, was used. The tissue was sent to Dovetail Genomics (Santa Cruz, CA) for Hi-C library preparation and sequencing on the Illumina HiSeq X platform.

4. De novo assembly

To generate a *de novo* assembly of long reads generated by SMRT sequencing, we conducted different pipelines including (i) Canu-Arrow-Pilon-Haplomerger2, (ii)

FALCON and FALCON-Unzip[4], and (iii) MaSuRCA[5]. The best assembly was generated by the (i) pipeline, which was used for further scaffolding. PacBio long reads were assembled using Canu (version 1.8)[6]. Next, all subreads were mapped using pbmm2 (ver. 1.0.0), and the assembly was polished using Arrow (ver. 2.3.3), provided in SMRT analysis 7.0.1 (Pacific Biosciences). Then, paired-end short reads were mapped to the assembly using BWA-MEM (ver. 0.7.15)[7] to correct erroneous bases using Pilon (ver. 1.23)[8]. In order to remove redundant contigs in the assembly, HaploMerger2 (ver. 20180603)[9] was used.

Using the PacBio RS II platform, we retrieved 6,634,481 subreads totaling 77,612,355,695 bases or approximately 67.5x coverage for a *P. fucata* genome size of 1.15Gbp[1] (Supplementary Table S1). A Canu assembly included 8,943 contigs totaling 1,765,389,74 bases. Because the Canu assembly contains a considerable number of duplicated sequences caused by high levels of heterozygosity in the *P. fucata* genome, we employed HaploMerger2[9] to obtain a haploid assembly with 3,129 contigs totaling 1,035,543,190 bases (Supplementary Table S2). The haplotype-merged assembly encoded 95.1% complete and single BUSCO genes and only 1.0% duplicated BUSCO genes (Supplementary Table S2), indicating that the redundant sequences were properly removed without a loss of unique sequences.

For the scaffolding step, the Long Ranger pipeline (ver. 2.2.2) provided by 10× Genomics[10] was used to apply 10X linked-read data. Then, 10X scaffolds and Dovetail Hi-C library reads were used in HiRise, which is a pipeline designed for using

proximity ligation data to scaffold genome assembly[11]. Hi-C assembly steps were performed by Dovetail Genomics.

The final MK assembly includes 414 scaffolds with an N50 length of 72.75 megabases (Supplementary Table S3). The sum of lengths of the 14 largest scaffolds (1,024 Mbp), ranging from 42 to 113 Mbp, comprised 98.6% of the all nucleotides of the assembly (1,039 Mbp). Because the number of chromosomes of *P. fucata* is 14[1], the present assembly is a chromosome-scale, haplotype-merged assembly. The genome contains 36,588 protein-coding loci with 96.2% BUSCO completeness and 0.9% duplicates (Supplementary Table S6), indicating that genome sequence redundancy derived from heterozygosity was collapsed in the final MK assembly.

5. Estimation of heterozygosity in inbred line

To estimate the level of heterozygosity in the inbred line, we conducted a k-mer-based statistical assessment with short read data. Approximately 60-fold paired-end Illumina short read sequences for *P. fucata* genome were analyzed using Jellyfish (version 2.2.10)[12] with different k-mer values ranging from 17 to 61. Next, the obtained frequency distribution of all k-mers was analyzed using GenomeScope[13]. Processed Illumina short reads were mapped to the MK genome assembly using BWA (version 0.7.15)[14]. Genotypes were retrieved using BCFtools (version 1.10) mpileup option[15]. A homozygous peak is observed at 60x coverage when k-mer=19 (Supplementary Figure S17).

Supplementary Figures

Fig. S1

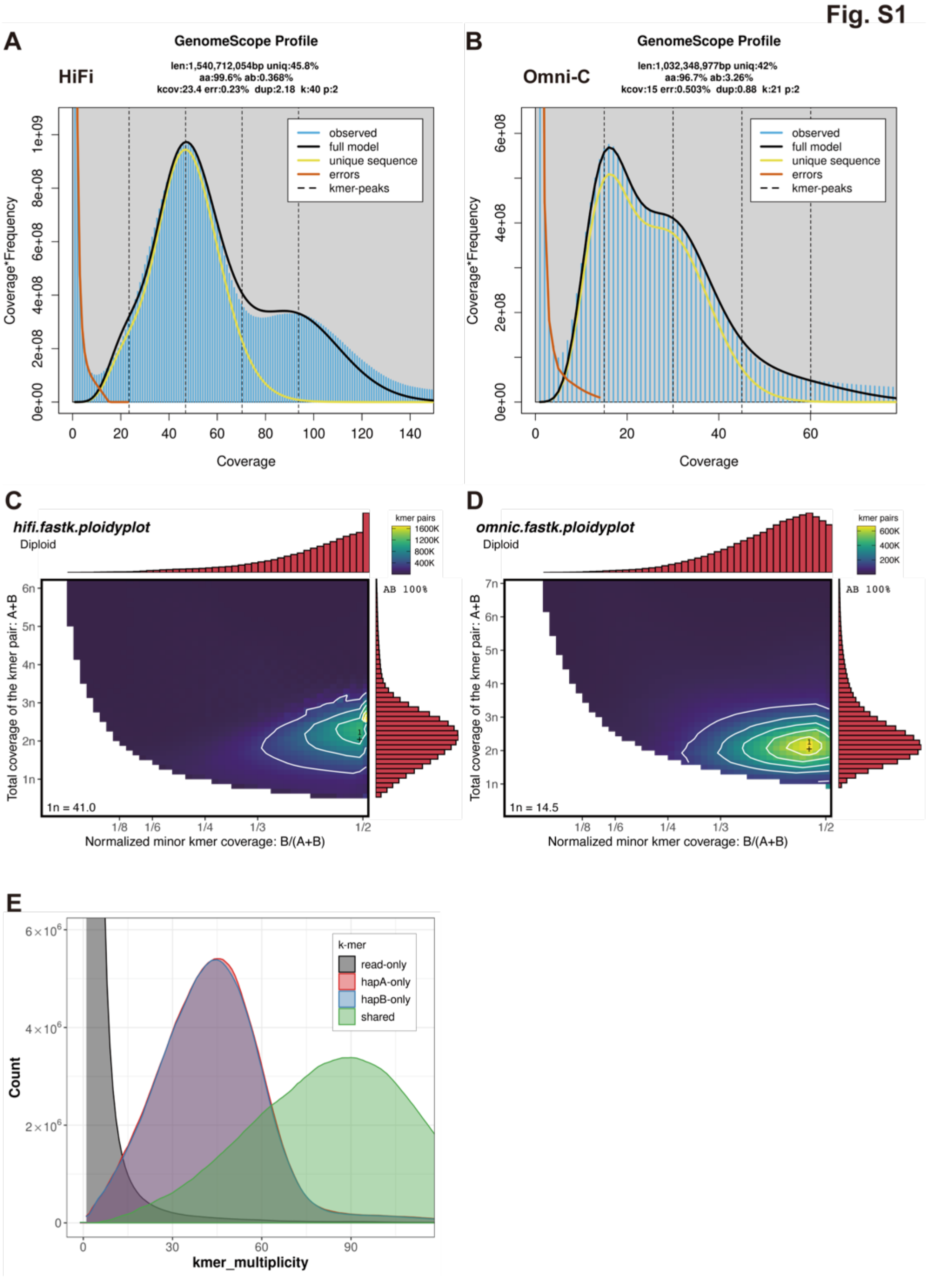

Supplementary Figure S1. K-mer analysis plots for sequencing reads and assembled scaffolds of the AI assembly. A–B) GeneScope plots of HiFi reads (A) and Omni-C reads (B). Note that the haploid coverage estimate in HiFi is wrong, although no peak actually exists at around 20x coverage. On the other hand, the curve fitting in Omni-C

seems correct and the estimated genome size is close to the value based on flow cytometry. C–D) PloidyPlots (improved version of SmudgePlot) of the HiFi reads (C) and the Omni-C reads (D). E) Merqury's spectra-asm plot with the two phased scaffold sets and the HiFi reads.

Fig. S2

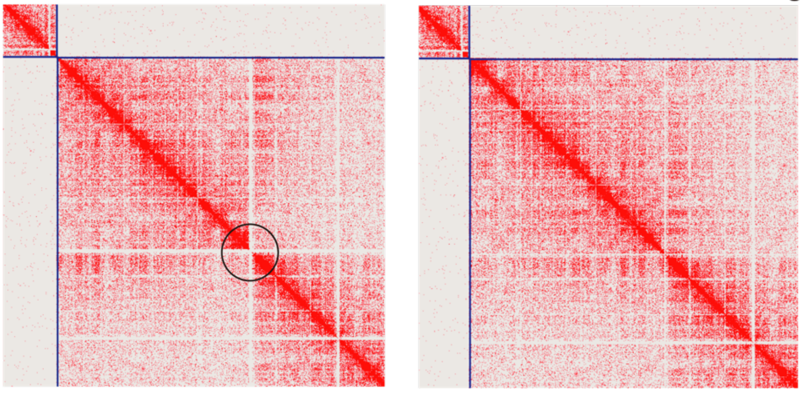

scaf 05b

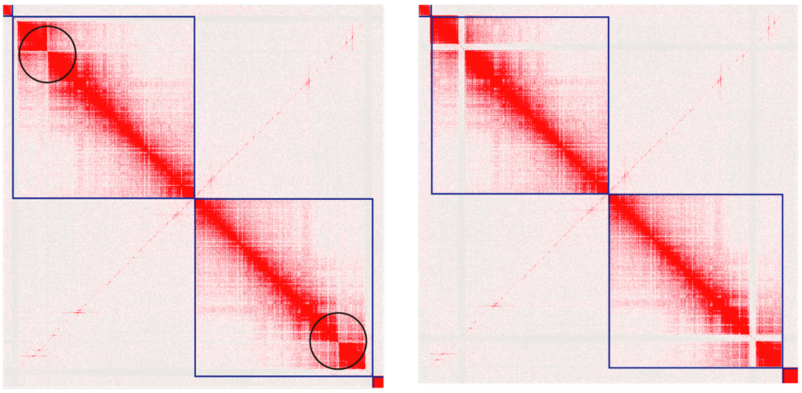

scaf 06a & scaf 06b (reverse complement)

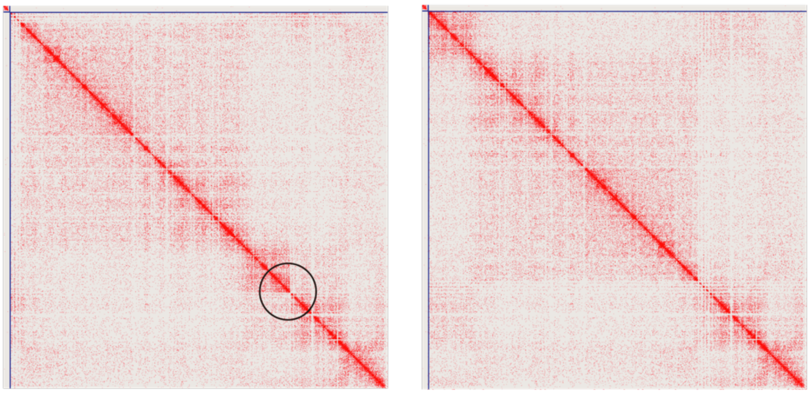

scaf 09b (reverse complement)

Fig. S2 (continued)

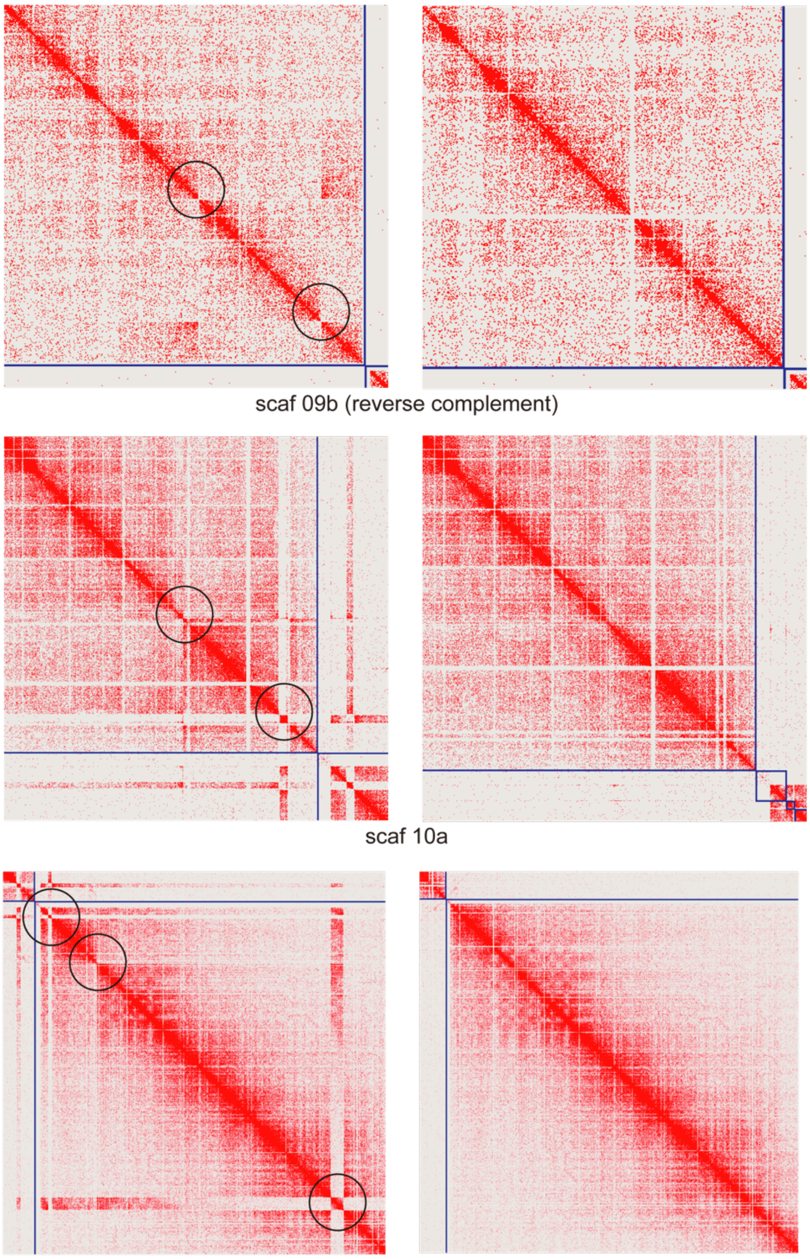

Supplementary Figure S2. Omni-C contact maps around manually curated locations before (left) and after (right) manual curation. Blue lines are boundaries between scaffolds. Gray areas indicate no unique mappings between the two locations due to repeats or misassemblies.

Fig. S3

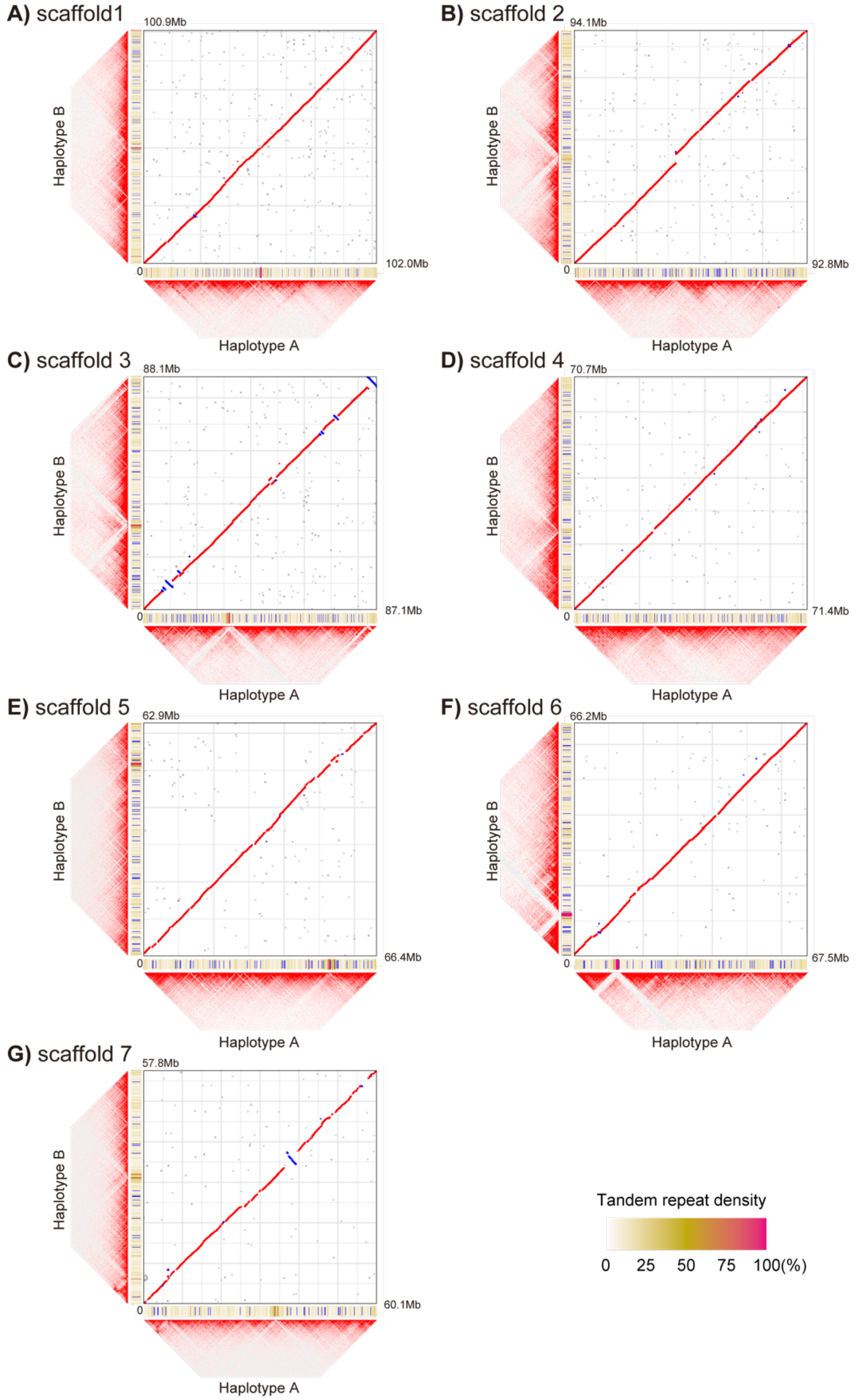

A) scaffold1
100.9Mb
102.0Mb
Haplotype B
Haplotype A
0
B) scaffold 2
94.1Mb
92.8Mb
Haplotype B
Haplotype A
0
C) scaffold 3
88.1Mb
87.1Mb
Haplotype B
Haplotype A
0
D) scaffold 4
70.7Mb
71.4Mb
Haplotype B
Haplotype A
0
E) scaffold 5
62.9Mb
66.4Mb
Haplotype B
Haplotype A
0
F) scaffold 6
66.2Mb
67.5Mb
Haplotype B
Haplotype A
0
G) scaffold 7
57.8Mb
60.1Mb
Haplotype B
Haplotype A
0
Tandem repeat density
0 25 50 75 100(%)

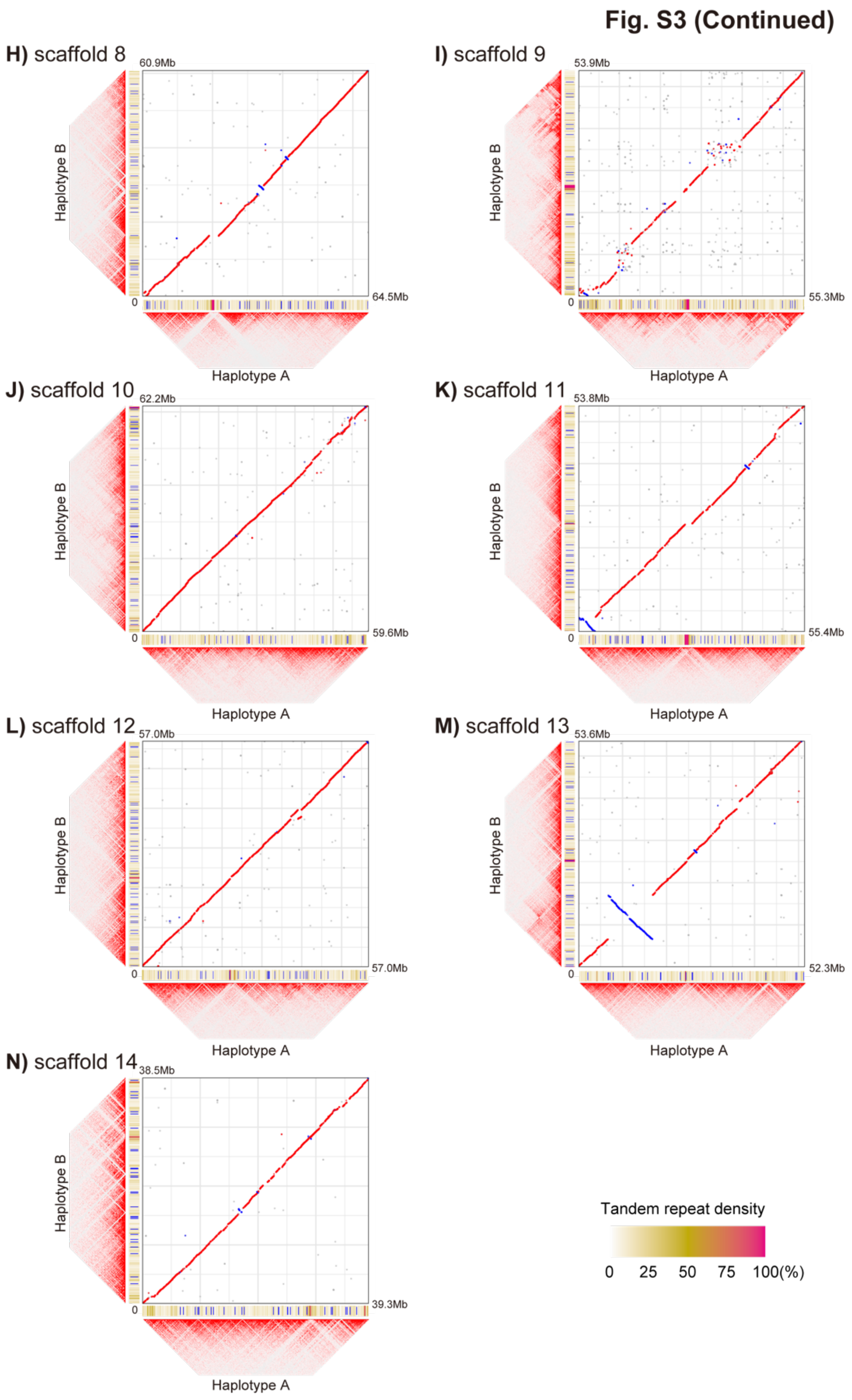

Supplementary Figure S3. Pairwise alignments of homologous chromosomes in haplotype A and B assemblies. Alignment dot plots were generated using MUMmer

3.23[16]. Aligned segments are represented as red (forward alignment) or blue (reverse alignment) dots. Heatmaps of tandem repeat density are shown along with x and y axes. Blue bars in the heatmaps indicate gap positions in the scaffolds. Omni-C contact maps for each scaffold are also shown at the x and y axes.

Fig. S4

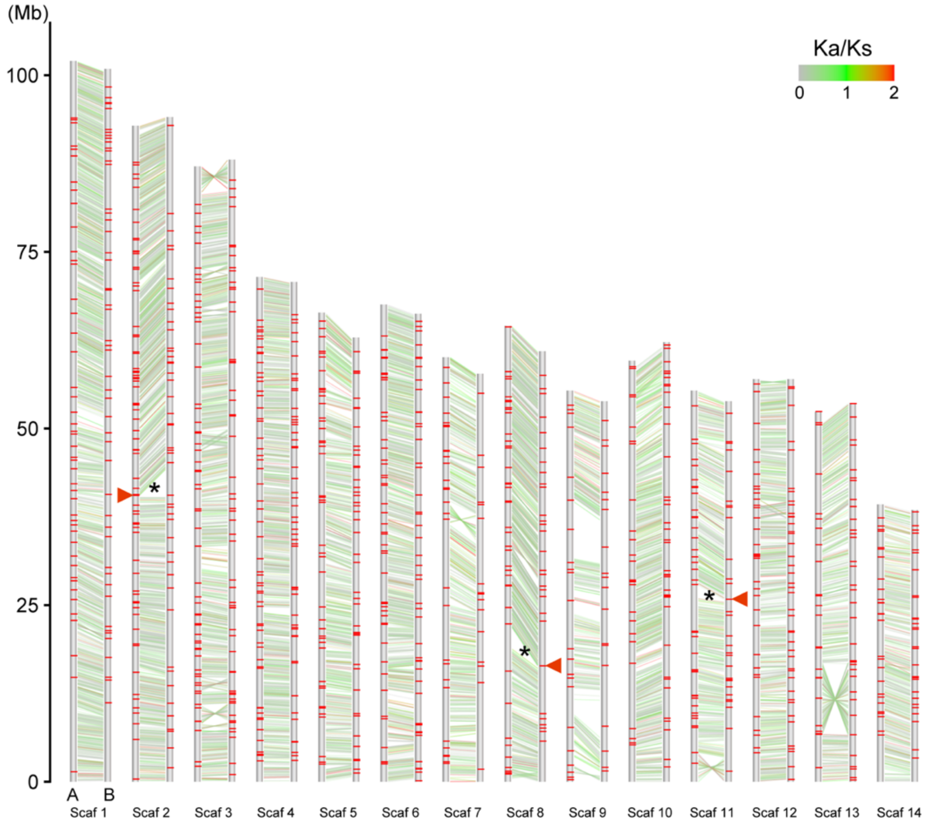

Supplementary Figure S4. Chromosomal scaffold length differences between haplotypes caused by unassembled sequences (gaps). Vertical gray bars indicate scaffold lengths and horizontal red lines indicate gap positions. Lines between scaffolds link homologous gene loci identified using MCScanX, colored based on Ka/Ks ratios. Length differences between haplotype A (left) and B (right) due to unassembled repetitive sequences are exemplified by asterisks in scaffolds 2, 8, and 11. In scaffold 2, a Helitron-rich region is found only in scaffold 2B, while the corresponding position in scaffold 2A was bridged by gaps (red arrowhead). In scaffolds 8 and 11, centromeric regions were fully sequenced and assembled only in haplotype A.

Fig. S5

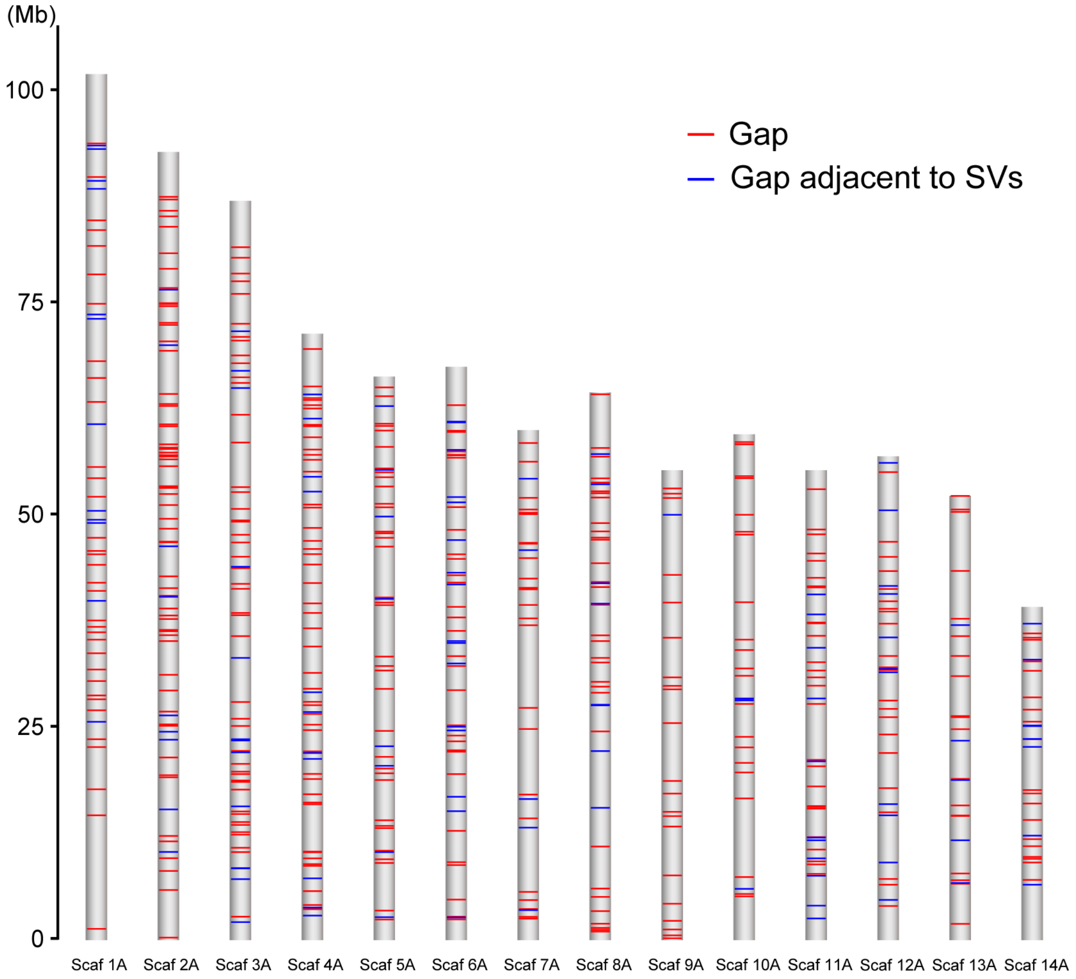

Supplementary Figure S5. Gap positions in haplotype A scaffolds. Vertical gray bars indicate scaffold lengths and horizontal lines in red or blue indicate gap positions. The gap positions in blue show that there are insertions or deletions within 500 bp of the gap position, indicating that the structural variants can be assembly errors. In the haplotype A scaffolds, 83 insertions (0.11% of total insertions) and 67 deletions (0.09%) are located within 500 bp of gaps. Therefore, 99.9% of insertions/deletions were supported by contiguous sequences without gaps built from HiFi reads.

Fig. S6

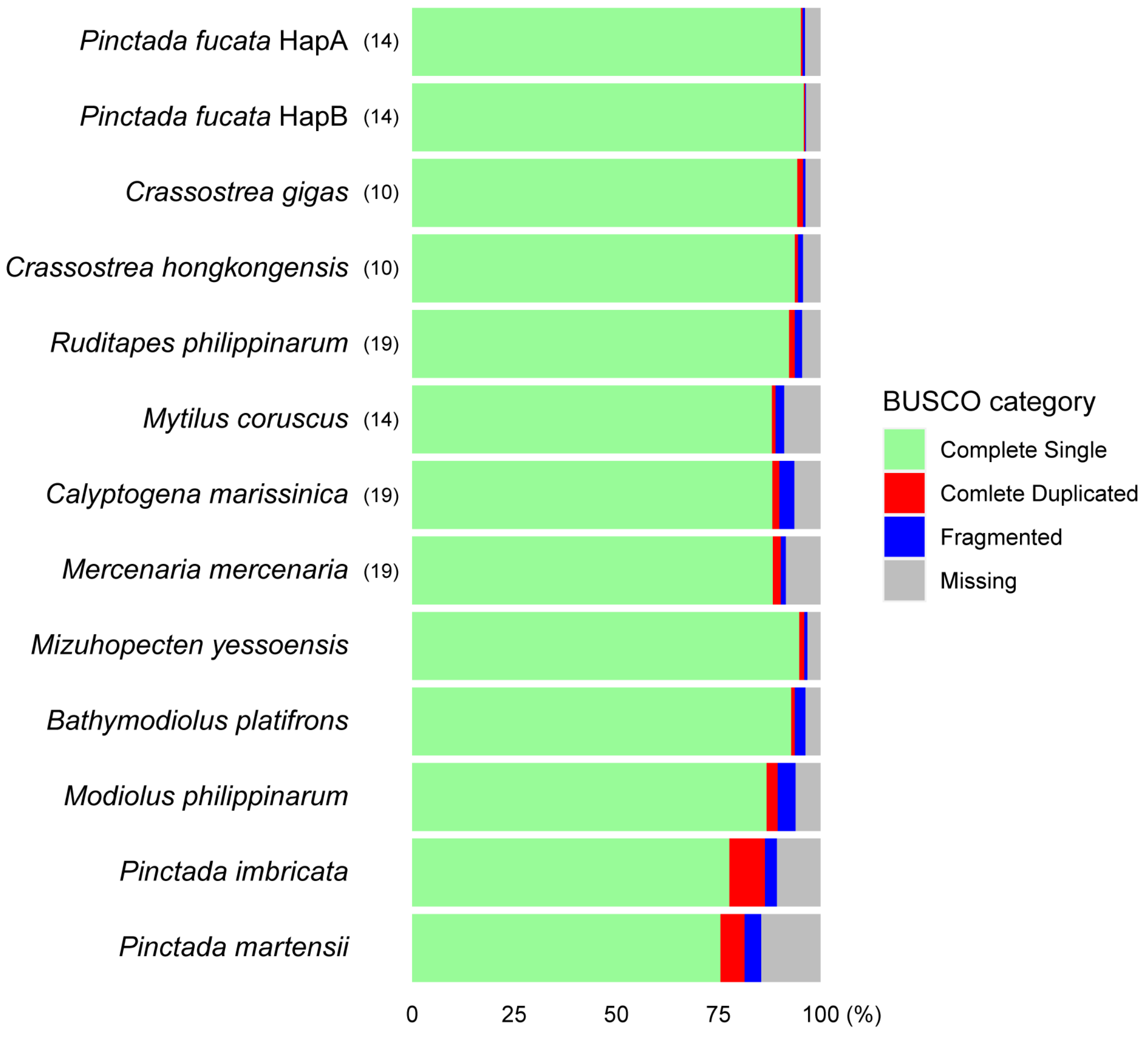

Supplementary Figure S6. BUSCO completeness of bivalve genomes. Genome assemblies were retrieved from NCBI. For chromosome-scale genome assemblies, the number of chromosomes is shown in parentheses at species names.

Fig. S7

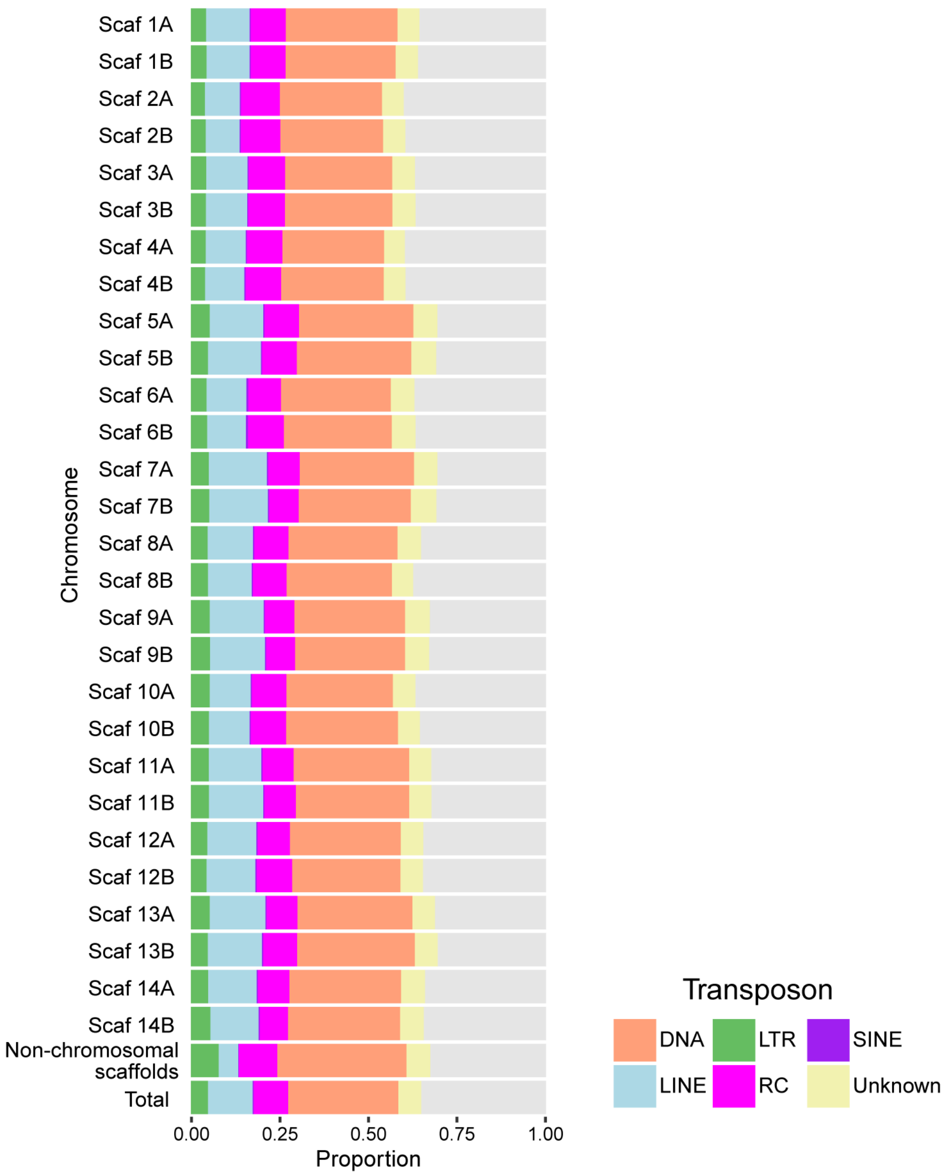

Supplementary Figure S7. TE contents of the *P. fucata* genome assembly for each scaffold. The two haplotypes contained similar levels of TEs.

Fig. S8

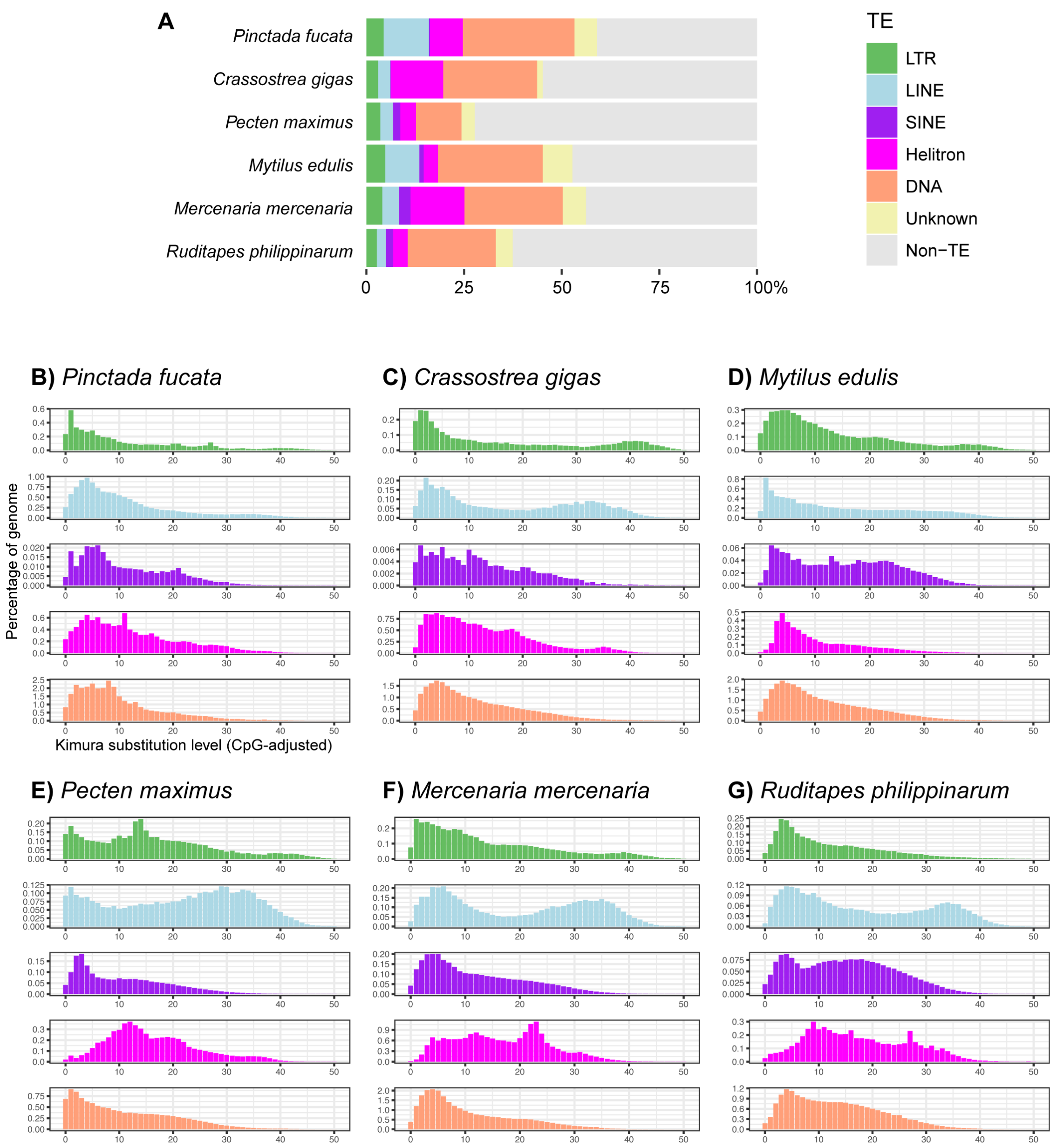

Supplementary Figure S8. TE contents and TE expansion history vary among bivalve genomes. The TE annotation pipeline of *P. fucata* was applied to selected bivalve genomes for comparison. A) TE contents of selected bivalve genomes. B-G) Distribution of Kimura substitution levels of TEs.

Fig. S9

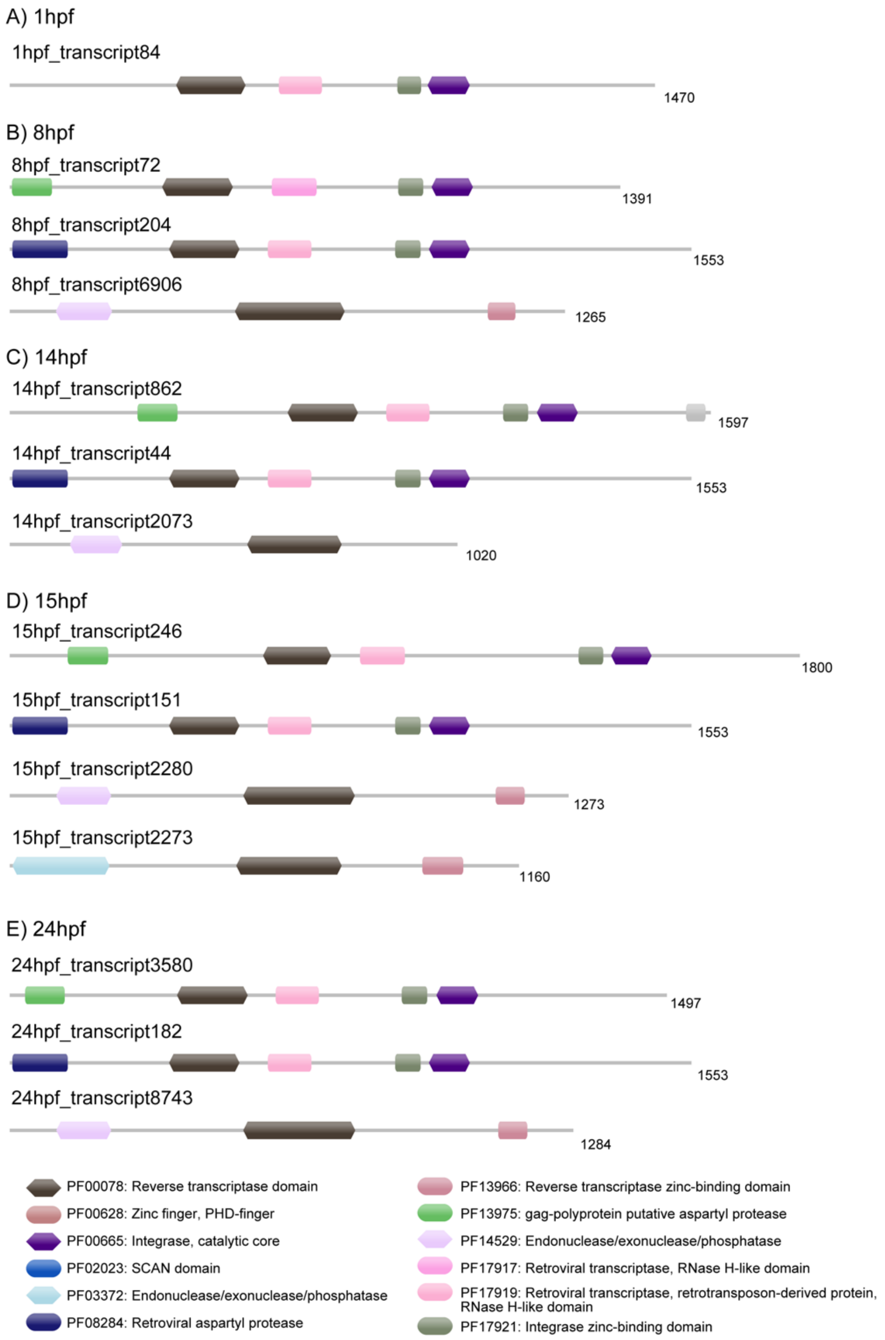

Supplementary Figure S9. Examples of domain architecture of retrotransposon-related gene products identified in Iso-seq read of early developmental stages.

Fig. S10

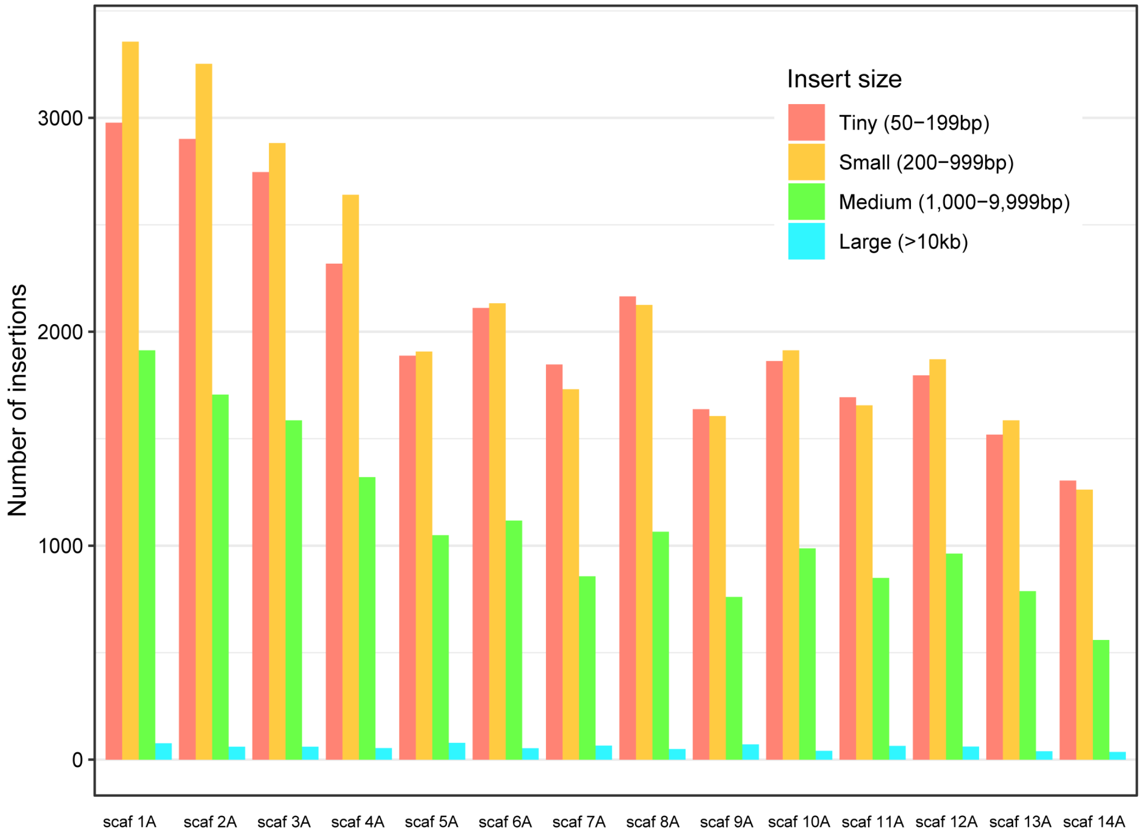

Supplementary Figure S10. The number of insertions in each chromosome.

Fig. S11

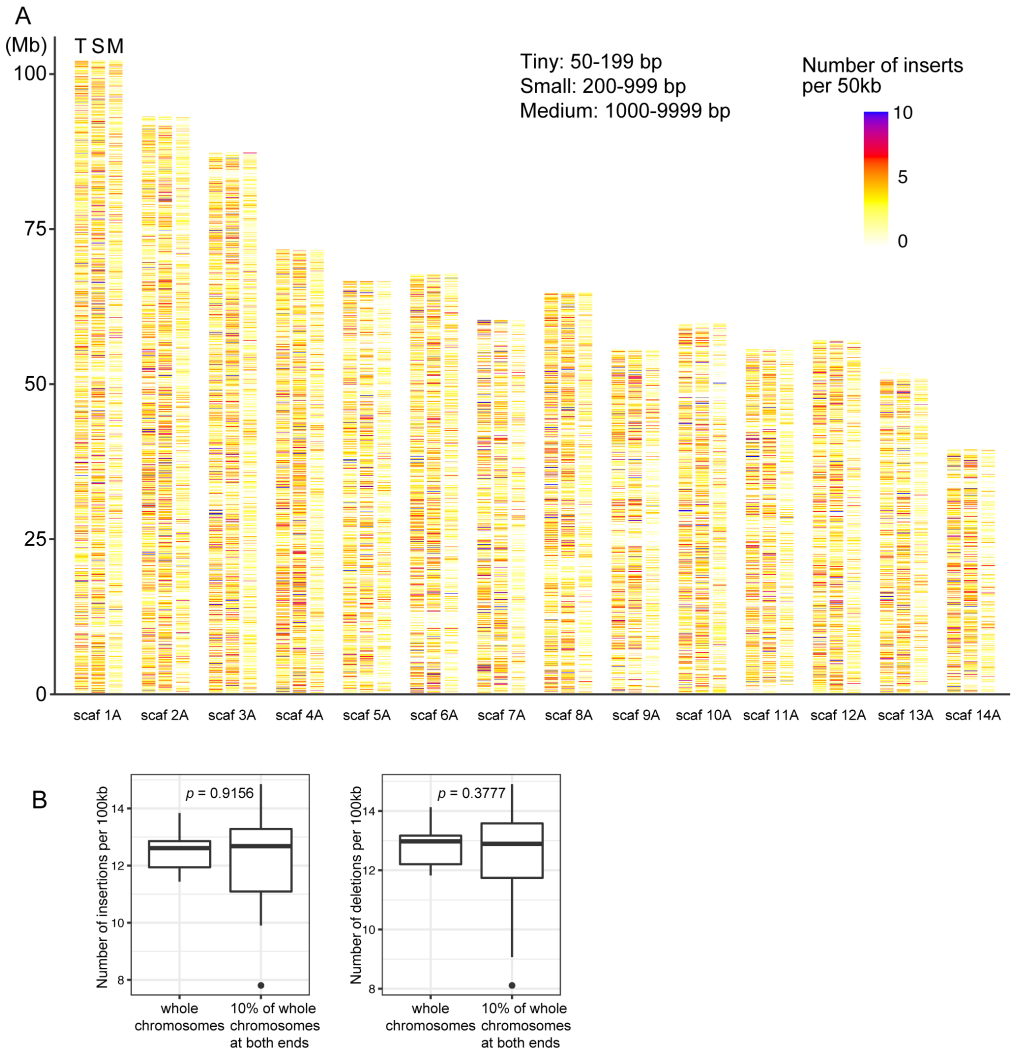

Supplementary Figure S11. Distributions of structural variants. A) Heatmap of insertion concentration indicates that they are distributed unevenly in chromosomal scaffolds. B) Average number of insertions (left) and deletions (right) per 100 kb in the whole chromosomal scaffolds and in 10% of the whole chromosomal ends. SVs are not significantly abundant in terminal regions of the chromosomes (Welch's t-test, $p > 0.05$).

Fig. S12

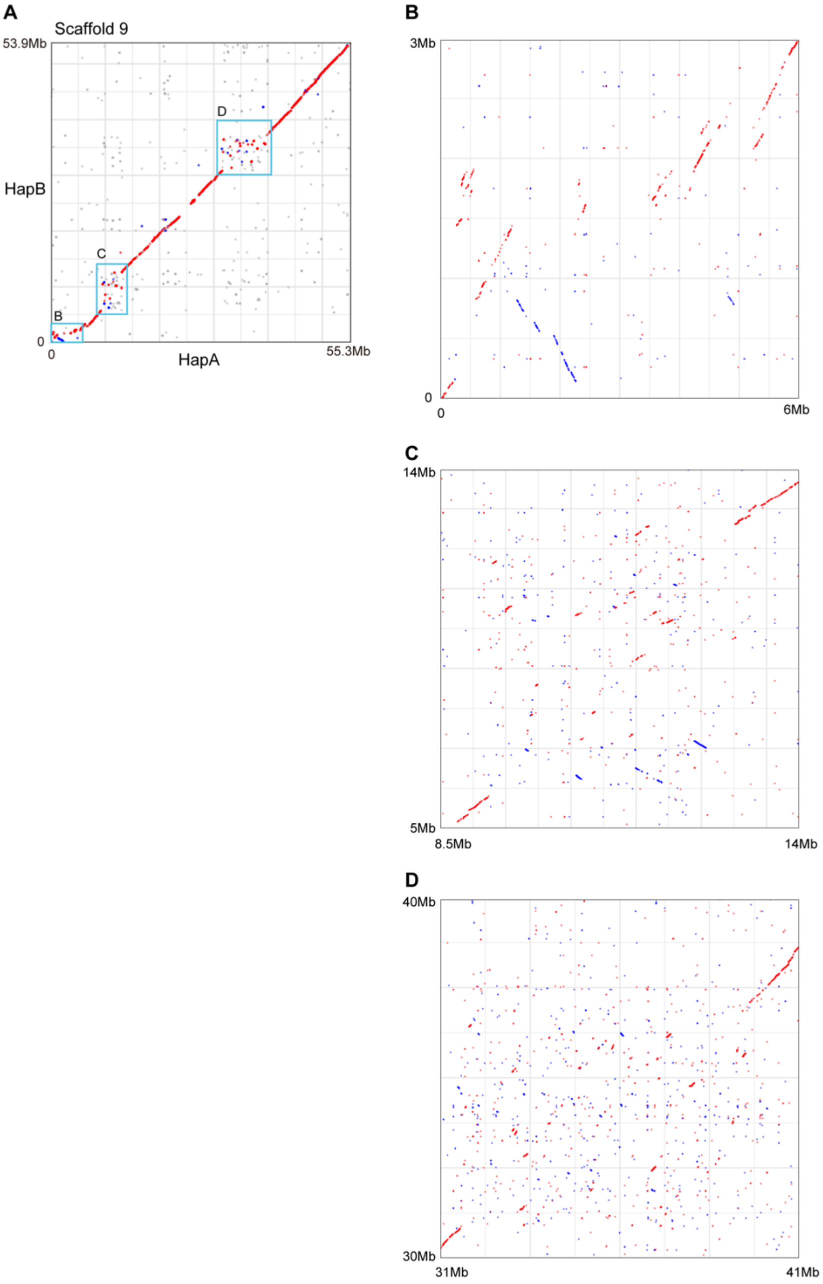

Supplementary Figure S12. Enlarged view of a pairwise alignment dotplot of non-syntenic regions in scaffold 9. A) Whole pairwise alignment of scaffold 9 haplotype A and B. B-D) Magnified figures of non-syntenic regions are indicated by cyan boxes in (A).

Fig. S13

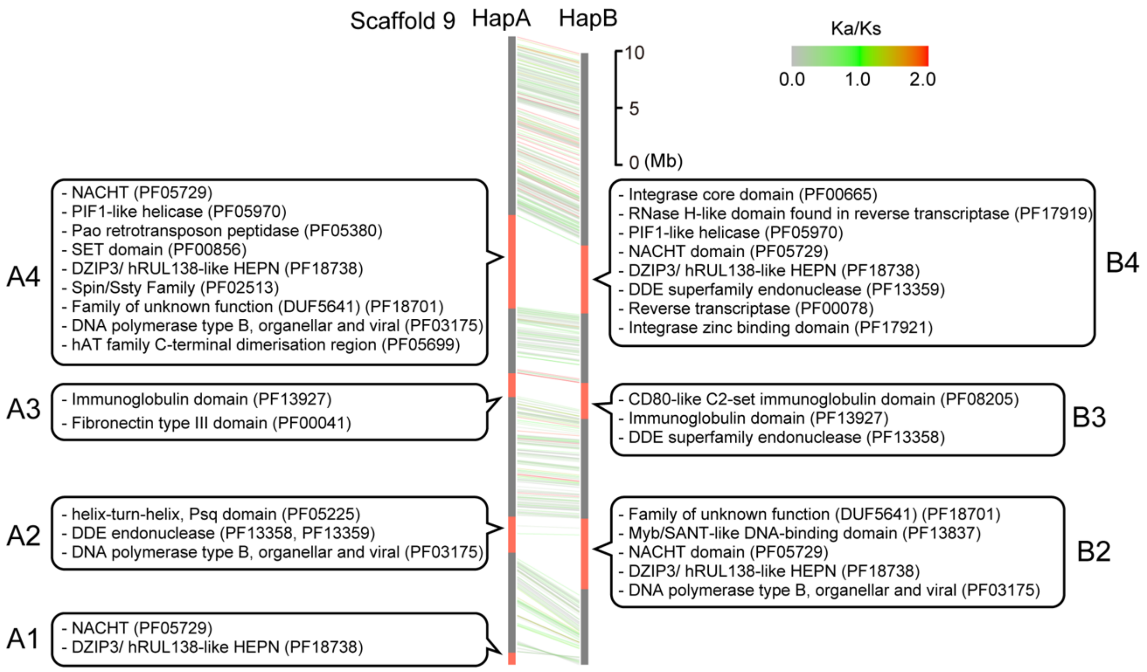

Supplementary Figure S13. Over-represented Pfam domains in non-syntenic regions of scaffold 9 were identified using a hypergeometric test (q-value < 0.001) adjusted using the Benjamini-Hochberg method.

Fig. S14

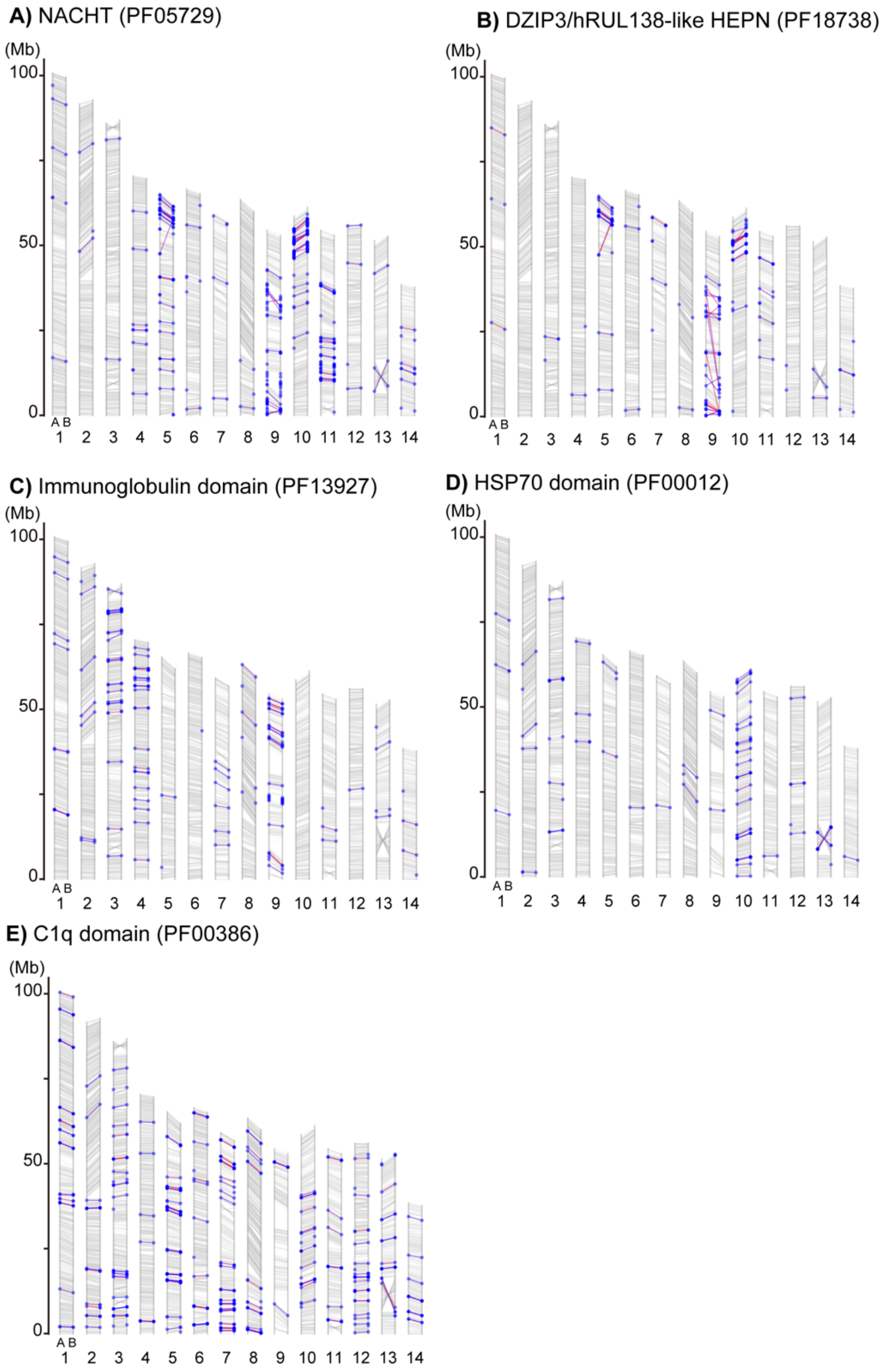

Supplementary Figure S14. Positions of gene models encoding functional domains related to innate defense system and response to environmental stressors. Gene positions are shown with blue dots. To identify allele pairs, nucleotide sequences of

haplotype A genes were Blastn-searched against haplotype B genes on the homologous chromosome. Best hit sequences with more than 90% sequence similarity are connected by lines. The color gradient of lines indicates sequence similarity from red (90%) to blue (100%). A) NACHT (PF05729). B) DZIP3/hRUL134-like HEPN (PF18738). C) Immunoglobulin domain (PF13927). D) Heat shock protein 70 (PF00012). E) C1q (PF00386). NACHT, DZIP3/hRUL134-like HEPN, and immunoglobulin DCP genes are clustered in non-syntenic regions. C1q and HSP70 (heat shock protein 70) gene loci are consistent between haplotypes and have no significant presence/absence variation.

Fig. S15

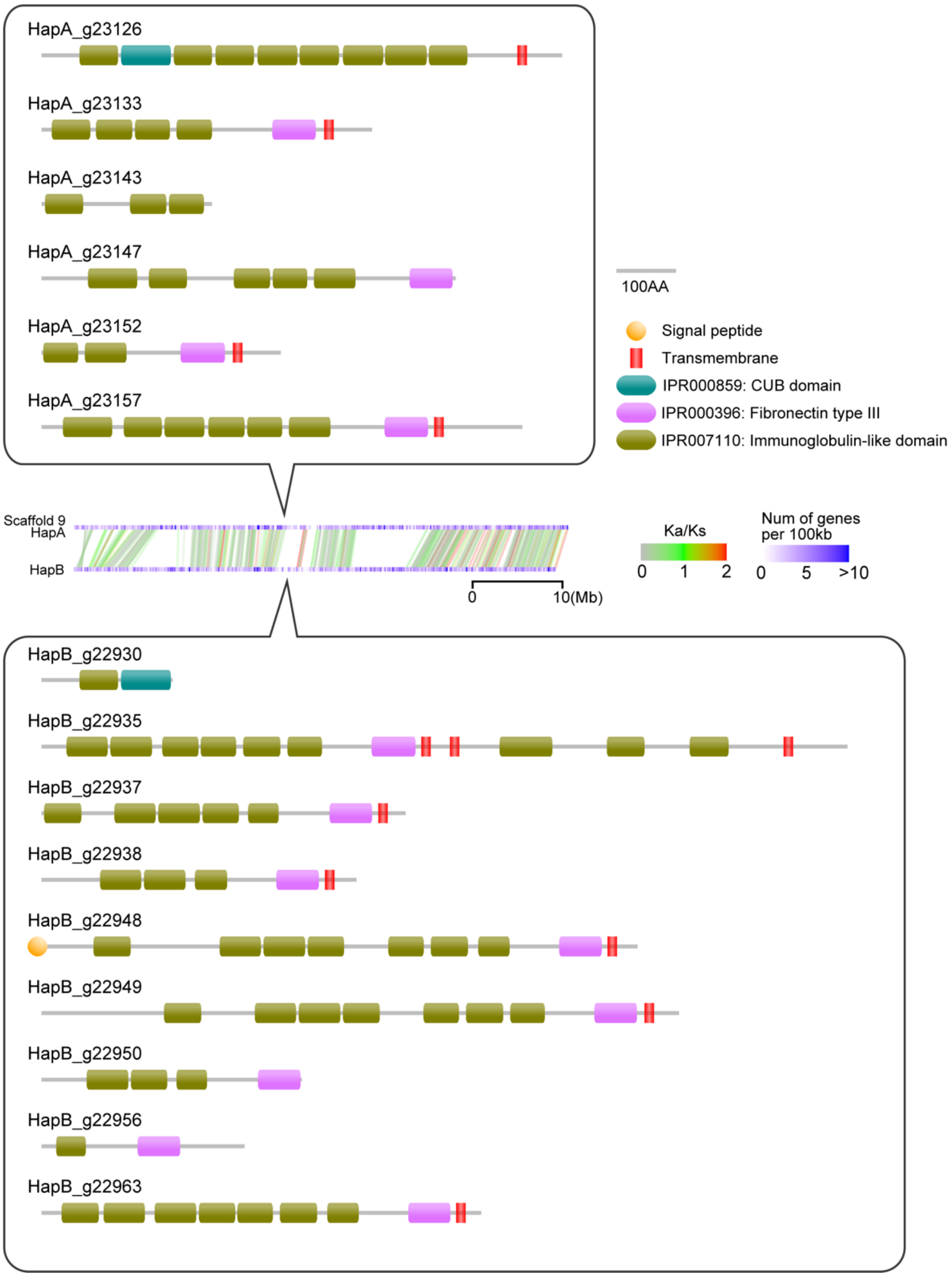

Supplementary Figure S15. Diverse Immunoglobulin repertoire in the non-syntenic region on scaffold 9.

Fig. S16

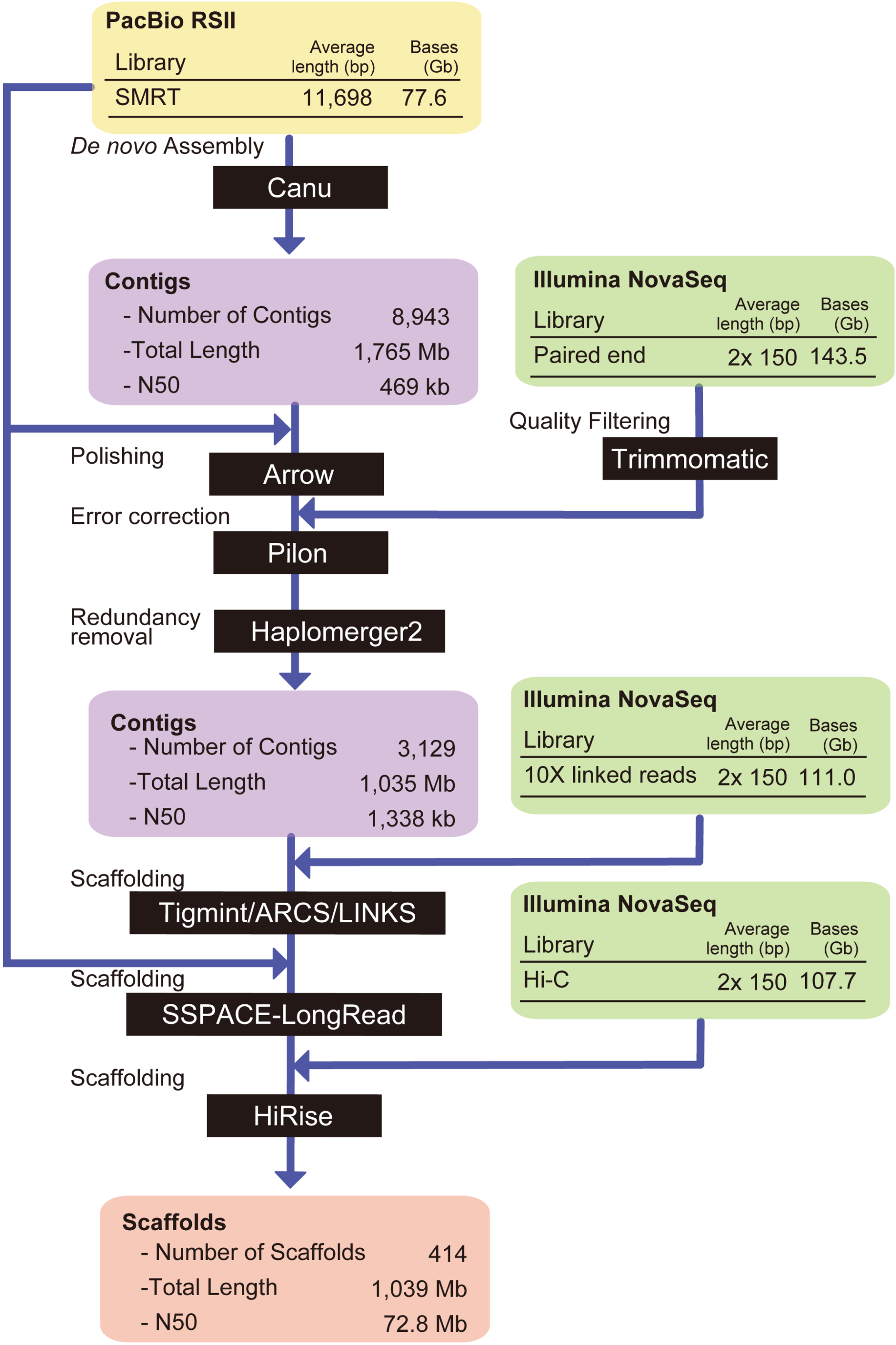

Supplementary Figure S16. Sequencing and assembly pipeline to produce the haplotype-merged MK genome assembly.

Fig. S17

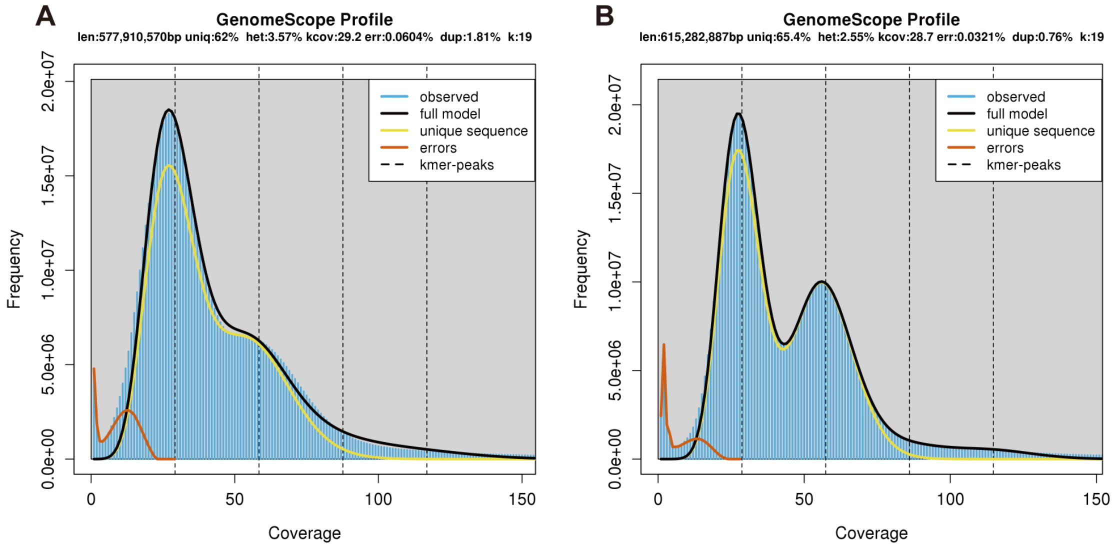

Supplementary Figure S17. GenomeScope k-mer analysis plots of the inbred line. Illumina short reads from the original individual (A) and from the third-generation individual (B). A primary peak at 30x coverage for heterozygous positions and a secondary peak at 60x coverage for homozygous positions are evident. The homozygous peak is more apparent in a third-generation individual, indicating that the heterozygosity rate was reduced after repetitive inbreeding.

Fig. S18

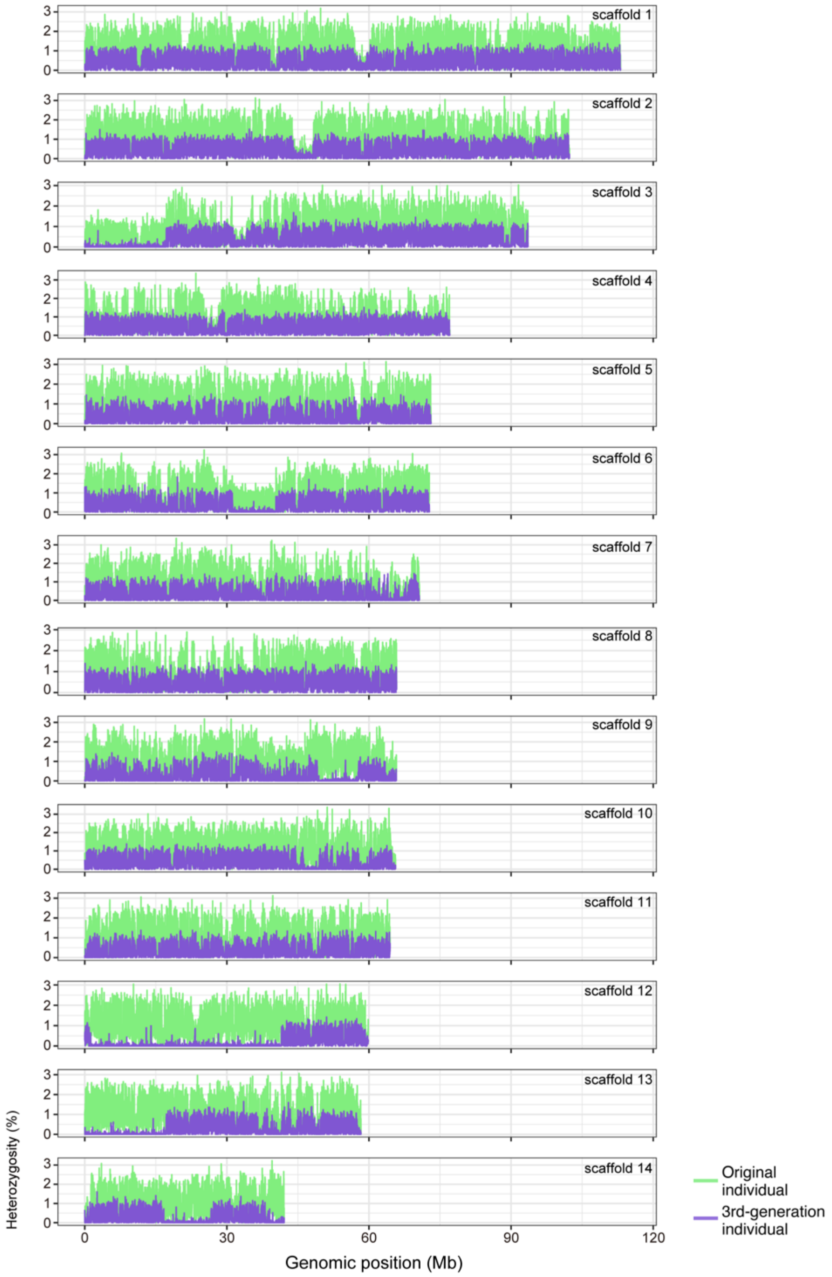

Supplementary Figure S18. The heterozygosity rate along the chromosomal scaffold. In this 3rd-generation individual, extremely reduced heterozygosity regions are found on scaffolds 3, 6, 9, 10, 12, 13, and 14, presumably due to autozygosity.

Supplementary Tables

Supplementary Table S1. Summary of *P. fucata* genome sequence data.

Assembly	Sequencer	Library type	Number of bases(Gbp)	Number of reads (million)	Average read length (bp)	Estimated coverage depth*	Accession
AI assembly (Haplotype-phased)	PacBio Sequel II	HiFi SMRTbell	79.3	3.8	20,869	69.0	DRA013512
	Illumina HiSeqX	Omni-C	39.8	132.5 x2	150	34.6	
MK assembly (Haplotype-merged)	Pacbio RSII	SMRTbell	77.6	6.6	11,698	67.5	DRA013511
	Illumina NovaSeq	1kb paired-end	143.5	478.5 x2	150	124.8	
		10x Chronium linked library	111.0	367.7 x2	151	96.6	
	Illumina HiSeqX	Hi-C	107.8	359.3 x2	150	93.7	

* Genome size of 1.15Gb

Supplementary Table S2. Summary of *P. fucata* genome assemblies.

	AI assembly (Haplotype-phased)			MK assembly (Haplotype-merged)
	All	Haplotype A	Haplotype B	
Number of contigs	2,064	-	-	3,129
contig N50 length (bp)	2,557,815	-	-	1,338,546
Number of scaffolds	1,001	14	14	414
scaffold N50 length (bp)	64,525,572	66,396,765	62,867,043	72,757,956
Total length (bp)	1,922,171,259	930,916,280	920,592,966	1,039,546,480
Sum of chromosomal scaffolds (bp)	1,851,509,246	-	-	1,024,560,685
Number of Gaps	1,102	565	533	3,400
Reliable block N50 (Mb)	2,078,788.00	-	-	1,003,834
k-mer QV	65.0	65.3	64.7	39.9
k-mer completeness	98.1	68.4	68.0	93.2
BUSCO benchmarking (metazoa_odb10)				
Complete (%)	96.3	95.6	96.2	96.1
Complete and single copy (%)	10.7	95.2	95.9	95.1
Complete and duplicated (%)	85.6	0.4	0.3	1.0
Fragmented (%)	0.2	0.6	0.2	0.8
Missing (%)	3.5	3.8	3.6	3.1

Supplementary Table S3. Sequence lengths and length differences between haplotypes.

Haplotype A					Haplotype B					Length difference (bp)
scaffold ID	Number of gaps	Length (bp)	Approximate length of short arm (Mbp)	Approximate length of long arm (Mbp)	scaffold ID	Number of gaps	Length (bp)	Approximate length of short arm (Mbp)	Approximate length of long arm (Mbp)	
Scaffold 1A	46	102,038,570	50.2	51.0	Scaffold 1B	50	100,905,477	49.8	50.7	1,133,093
Scaffold 2A	70	92,860,333	-	-	Scaffold 2B	46	94,092,351	-	-	1,232,018
Scaffold 3A	60	87,098,167	32.1	54.8	Scaffold 3B	55	88,058,079	31.7	56.2	959,912
Scaffold 4A	57	71,437,201	-	-	Scaffold 4B	53	70,740,401	-	-	696,800
Scaffold 5A	44	66,396,765	13.0	53.0	Scaffold 5B	42	62,867,043	10.6	51.7	3,529,722
Scaffold 6A	47	67,553,417	-	-	Scaffold 6B	46	66,243,863	-	-	1,309,554
Scaffold 7A	29	60,090,080	-	-	Scaffold 7B	17	57,759,605	-	-	2,330,475
Scaffold 8A	40	64,525,572	19.7	44.1	Scaffold 8B	29	60,944,300	-	-	3,581,272
Scaffold 9A	22	55,353,753	26.5	28.1	Scaffold 9B	21	53,860,184	25.7	27.4	1,493,569
Scaffold 10A	28	59,595,499	-	-	Scaffold 10B	40	62,212,318	-	-	2,616,819
Scaffold 11A	38	55,365,116	26.1	28.4	Scaffold 11B	30	53,849,958	-	-	1,515,158
Scaffold 12A	33	57,001,201	-	-	Scaffold 12B	42	56,990,428	-	-	10,773
Scaffold 13A	24	52,343,680	-	-	Scaffold 13B	34	53,606,897	-	-	1,263,217
Scaffold 14A	27	39,256,926	-	-	Scaffold 14B	28	38,462,062	-	-	794,864

Supplementary Table S4. Statistics of RNA-seq reads after Trimmomatic filtering.

Developmental stage	Number of bases(bp)	Number of reads
Unfertilized egg	4,781,306,940	19,311,079
Fertilized egg	9,049,673,698	36,266,744
0.5 hpf	5,363,581,348	21,453,078
1.0 hpf	4,216,964,603	16,936,582
1.5 hpf	4,559,851,143	18,268,546
2.0 hpf	4,054,971,113	16,431,379
3.0 hpf	6,344,803,282	25,646,015
4.0 hpf	4,205,404,392	16,988,106
5.0 hpf	3,661,662,525	14,945,200
6.0 hpf	4,725,582,628	19,135,842
7.0 hpf	4,670,653,162	18,789,478
8.0 hpf	4,713,702,484	19,025,280
24.0 hpf	4,531,887,822	18,206,752
15 dpf	4,793,191,685	19,278,898
20 dpf	4,330,293,318	17,449,028

hpf: Hours post-fertilization

dpf: Days post-fertilization

Supplementary Table S5. Statistics of Iso-Seq reads after clustering and polishing using isoseq3.

Adult tissue / Developmental stage	Number of bases (bp)	Number of isoforms	Average length (bp)
Whole mantle (individual 1)	89,565,688	23,893	3748.616
Whole mantle (individual 2)	87,770,434	23,600	3719.086
Whole mantle (individual 3)	85,996,375	26,531	3241.354
Mantle edge	47,412,729	14,247	3327.91
Mantle pallium	119,597,497	33,401	3580.656
Adductor muscle	72,047,624	16,334	4410.899
Gill	96,403,863	27,032	3566.287
Byssus gland	90,603,631	24,279	3731.769
1 hpf	62,154,272	22,384	2776.728
8 hpf	90,370,320	23,381	3865.118
14 hpf	65,904,521	22,559	2921.429
15 hpf	83,632,224	22,783	3670.817
24 hpf	99,928,408	24,999	3997.296

hpf: Hours post-fertilization

Supplementary Table S6. Summary of *P. fucata* gene models.

	Genome assembly		
	AI haplotype A	AI haplotype B	MK
Number of gene models	32,938	32,759	36,588
Number of transcripts	41,208	41,149	44,399
Average length (bp)	15,842	15,923	15,742
BUSCO benchmarking			
metazoa_odb10			
Complete	94.6%	96.2%	96.2%
Complete and single copy	93.7%	95.4%	95.3%
Complete and duplicated	0.9%	0.8%	0.9%
Fragmented	3.0%	2.2%	2.6%
Missing	2.4%	1.6%	1.2%
mollusca_odb10			
Complete	93.5%	93.2%	92.6%
Complete and single copy	92.3%	92.0%	91.1%
Complete and duplicated	1.2%	1.2%	1.5%
Fragmented	1.4%	1.5%	2.1%
Missing	5.1%	5.3%	5.3%

Supplementary Table S7. Repeat elements in the *P. fucata* genome AI assembly.

Retrotransposons (Class I)		
LTR	Gypsy	2.69%
	Copia	0.28%
	DIRS	0.15%
	BEL	0.08%
	Ngaro	0.06%
	ERV1	0.01%
	ERVL	0.01%
	ERV	0.01%
	unclassified	1.14%
	total	4.43%
LINE	Penelope	10.34%
	L1	0.40%
	RTE	0.33%
	CR1	0.31%
	L2	0.10%
	Proto2	0.05%
	I	0.03%
	unclassified	0.02%
	total	11.58%
SINE	tRNA	0.15%
	MIR	0.08%
	ID	0.03%
	B4	0.03%
	total	0.28%
DNA transposons (Class II)		
	Helitron	8.42%
	TcMar	6.49%
	hAT	5.26%
	nMITE	4.52%
	MITE	4.28%
	Mutator	1.21%
	Crypton	0.94%
	PIF	0.85%
	Maverick	0.59%
	Harbinger	0.56%
	Zator	0.32%
	Academ	0.26%
	CACTA	0.18%
	CMC	0.15%
	Sola	0.11%
	Ginger	0.10%
	IS3EU	0.08%
	Kolobok	0.07%
	PiggyBac	0.03%
	MULE	0.02%
	Merlin	0.01%
	P	0.01%
	unclassified	2.56%
	total	37.01%
unknown		
	total	5.70%
Tandem repeats		
	microsatellite (1-6bp)	0.36%
	7-10bp	0.13%
	minisatellite (11-100bp)	4.76%
	satellite (>100bp)	2.50%
	total	7.75%

Supplementary Table S8. Number of Iso-Seq transcripts encoding reverse transcriptase and transposase domains.

Adult tissue / Developmental stage	Reverse transcriptase / RNase H Pfam ID			Transposase Pfam ID					
	PF00078	PF17917	PF17919	PF01359	PF01498	PF02992	PF03221	PF10551	PF12762	PF13843
Whole mantle (individual 1)	117	2	15	0	3	1	2	0	0	1
Whole mantle (individual 2)	177	9	12	1	6	0	1	1	0	0
Whole mantle (individual 3)	137	7	10	0	0	1	2	3	2	1
Mantle edge	56	2	0	0	1	1	1	0	0	1
Mantle pallium	163	10	21	0	4	1	3	1	0	3
Adductor muscle	55	3	6	0	2	1	0	0	0	0
Gill	108	10	18	0	1	1	1	1	0	1
Byssus gland	88	1	12	0	4	1	1	1	0	1
1 hpf	4	0	2	0	0	0	2	0	0	0
8 hpf	152	3	29	0	2	2	4	2	1	0
14 hpf	87	8	17	0	1	2	0	0	0	1
15 hpf	90	5	19	0	2	2	1	0	0	0
24 hpf	215	38	32	0	1	2	0	1	0	0

hpf: Hours post-fertilization

Supplementary Table S9. Insertion and deletion variants in chromosomal scaffolds.

	Size	Number of SVs	Base pairs	% genome
Insertion	Tiny (50-199bp)	28,767	3,085,895	0.33%
	Small (200-999bp)	29,920	13,883,093	1.49%
	Medium (1,000-9,999bp)	15,518	49,474,360	5.31%
	Large (>10kb)	807	9,700,360	1.04%
	Total	75,012	76,143,708	8.18%
Deletion	Tiny (50-199bp)	31,058	2,989,015	0.35%
	Small (200-999bp)	26,606	12,282,644	1.29%
	Medium (1,000-9,999bp)	13,300	43,164,934	4.61%
	Large (>10kb)	1,567	33,928,784	3.69%
	Total	72,531	92,515,013	9.94%

Supplementary Table S10. Number of structural variants in each scaffold.

Scaffold ID	Whole scaffold					10% of whole scaffold at both ends	
	Number of insertions	Total length of insertions (bp)	Number of Deletions	Total length of deletions (bp)	Difference between ins. and del. in length (bp)	Number of insertions	Number of Deletions
Scaffold 1A	12,849	8,892,367	13,275	10,952,365	2,059,998	2637 (20.5%)	2699 (20.3%)
Scaffold 2A	12,218	7,870,132	12,684	9,632,049	1,761,917	2218 (18.2%)	2450 (19.3%)
Scaffold 3A	11,162	7,188,119	11,308	8,677,044	1,488,925	2214 (19.8%)	2203 (19.5%)
Scaffold 4A	9,888	6,137,837	10,094	7,343,896	1,206,059	1997 (20.2%)	1973 (19.5%)
Scaffold 5A	7,672	5,576,735	8,000	7,163,872	1,587,137	1539 (20.1%)	1549 (19.4%)
Scaffold 6A	8,470	5,469,689	8,807	6,937,185	1,467,496	1787 (21.1%)	1835 (20.8%)
Scaffold 7A	7,129	4,840,663	7,211	6,153,204	1,312,541	1467 (20.6%)	1414 (19.6%)
Scaffold 8A	8,348	5,309,357	8,564	5,882,442	573,085	1729 (20.7%)	1749 (20.4%)
Scaffold 9A	6,333	4,559,024	6,546	4,826,501	267,477	1236 (19.5%)	1247 (19.0%)
Scaffold 10A	7,551	4,664,316	7,754	5,982,940	1,318,624	1414 (18.7%)	1498 (19.3%)
Scaffold 11A	6,743	4,662,158	7,016	5,875,699	1,213,541	1232 (18.3%)	1301 (18.5%)
Scaffold 12A	7,278	5,037,958	7,417	6,081,844	1,043,886	1569 (21.6%)	1474 (19.9%)
Scaffold 13A	6,084	4,067,164	6,284	4,959,506	892,342	1134 (18.6%)	1185 (18.9%)
Scaffold 14A	5,053	3,099,253	5,191	3,660,729	561,476	923 (18.3%)	952 (18.3%)

Supplementary Table S11. Enriched Pfam domains in the non-syntenic region in scaffold 9 (A1 in Supplementary Fig. S13).

Pfam ID	Name	q-value
PF05729	NACHT domain	0.00013768
PF18738	DZIP3/ hRUL138-like HEPN	0.00027341
PF05380	Pao retrotransposon peptidase	0.01141294
PF01920	Prefoldin subunit	0.01390336
PF05699	hAT family C-terminal dimerisation region	0.04819279
PF08477	Ras of Complex, Roc, domain of DAPkinase	0.05619715
PF00754	F5/8 type C domain	0.06353693
PF02338	OTU-like cysteine protease	0.06748129
PF14529	Endonuclease-reverse transcriptase	0.06794811
PF00041	Fibronectin type III domain	0.07933969
PF18701	Family of unknown function (DUF5641)	0.08488343
PF00096	Zinc finger, C2H2 type	0.17826069

Supplementary Table S12. Enriched Pfam domains in the non-syntenic region in scaffold 9 (A2 in Supplementary Fig. S13).

Pfam ID	Name	q-value
PF05225	helix-turn-helix, Psq domain	0.001431759
PF13359	DDE superfamily endonuclease	0.002399241
PF13358	DDE superfamily endonuclease	0.003341922
PF03175	DNA polymerase type B, organellar and viral	0.004180309
PF03184	DDE superfamily endonuclease	0.007048741
PF12560	RAG1 importin binding	0.012565174
PF05970	PIF1-like helicase	0.012646121
PF16064	Domain of unknown function (DUF4806)	0.018816866
PF01498	Transposase	0.041610151
PF02229	Transcriptional Coactivator p15 (PC4)	0.062075632
PF03732	Retrotransposon gag protein	0.08955731
PF00619	Caspase recruitment domain	0.094100862
PF14893	PNMA	0.098703513
PF00856	SET domain	0.101526746
PF14214	Helitron helicase-like domain at N-terminus	0.102724929
PF13472	GDSL-like Lipase/Acylhydrolase family	0.102724929
PF18701	Family of unknown function (DUF5641)	0.161284665
PF13975	gag-polyprotein putative aspartyl protease	0.180287419
PF00665	Integrase core domain	0.220726339
PF05729	NACHT domain	0.240026529
PF00098	Zinc knuckle	0.262934413
PF17921	Integrase zinc binding domain	0.265597899

Supplementary Table S13. Enriched Pfam domains in the non-syntenic region in scaffold 9 (A3 in Supplementary Fig. S13).

Pfam ID	Name	q-value
PF13927	Immunoglobulin domain	2.75E-05
PF00041	Fibronectin type III domain	3.48E-05
PF07679	Immunoglobulin I-set domain	0.001834197
PF00078	Reverse transcriptase (RNA-dependent DNA polymerase)	0.003574165
PF04843	Herpesvirus tegument protein, N-terminal conserved region	0.01436139
PF12012	Domain of unknown function (DUF3504)	0.014558667
PF14214	Helitron helicase-like domain at N-terminus	0.026173503
PF05970	PIF1-like helicase	0.028535823
PF17919	RNase H-like domain found in reverse transcriptase	0.030956291

Supplementary Table S14. Enriched Pfam domains in the non-syntenic region in scaffold 9 (A4 in Supplementary Fig. S13).

Pfam ID	Name	q-value
PF05729	NACHT domain	1.00E-08
PF05970	PIF1-like helicase	2.19E-06
PF05380	Pao retrotransposon peptidase	3.83E-05
PF00856	SET domain	0.00097923
PF18738	DZIP3/ hRUL138-like HEPN	0.00121778
PF02513	Spin/Ssty Family	0.00137402
PF18701	Family of unknown function (DUF5641)	0.00142118
PF03175	DNA polymerase type B, organellar and viral	0.00217852
PF05699	hAT family C-terminal dimerisation region	0.00278338
PF17921	Integrase zinc binding domain	0.00633896
PF14214	Helitron helicase-like domain at N-terminus	0.00885849
PF05485	THAP domain	0.00901348
PF02992	Transposase family tnp2	0.00931477
PF04843	Herpesvirus tegument protein, N-terminal conserved region	0.01649428
PF00665	Integrase core domain	0.03072473
PF06869	Protein of unknown function (DUF1258)	0.04092981
PF13613	Helix-turn-helix of DDE superfamily endonuclease	0.05414989
PF03184	DDE superfamily endonuclease	0.05858237
PF13873	Myb/SANT-like DNA-binding domain	0.06036919
PF02017	CIDE-N domain	0.06505488
PF00628	PHD-finger	0.0756418
PF13843	Transposase IS4	0.07850718
PF10545	Alcohol dehydrogenase transcription factor Myb/SANT-like	0.07850718
PF01771	Viral alkaline exonuclease	0.08078229
PF09588	YqaJ-like viral recombinase domain	0.08515662
PF04218	CENP-B N-terminal DNA-binding domain	0.08560069
PF13359	DDE superfamily endonuclease	0.08822964
PF00270	DEAD/DEAH box helicase	0.08940573
PF00292	'Paired box' domain	0.08954564
PF17919	RNase H-like domain found in reverse transcriptase	0.09068529
PF00385	Chromo (CHRromatin Organisation MOdifier) domain	0.09073854
PF05225	helix-turn-helix, Psq domain	0.09965454
PF10523	BEN domain	0.09965454
PF03221	Tc5 transposase DNA-binding domain	0.11181345
PF14291	Domain of unknown function (DUF4371)	0.12016065
PF00271	Helicase conserved C-terminal domain	0.12779656
PF02229	Transcriptional Coactivator p15 (PC4)	0.14054007
PF00929	Exonuclease	0.14714701
PF03732	Retrotransposon gag protein	0.2065115
PF00078	Reverse transcriptase (RNA-dependent DNA polymerase)	0.22641487
PF00619	Caspase recruitment domain	0.2285833
PF01026	TatD related DNase	0.2553306
PF13472	GDSL-like Lipase/Acylhydrolase family	0.25923418
PF14529	Endonuclease-reverse transcriptase	0.26132176
PF13920	Zinc finger, C3HC4 type (RING finger)	0.29022578
PF00098	Zinc knuckle	0.33001414
PF00096	Zinc finger, C2H2 type	0.37243642
PF13975	gag-polyprotein putative aspartyl protease	0.4747128
PF00147	Fibrinogen beta and gamma chains, C-terminal globular domain	0.5134518

Supplementary Table S15. Enriched Pfam domains in the non-syntenic region in scaffold 9 (B2 in Supplementary Fig. S13).

Pfam ID	Name	q-value
PF18701	Family of unknown function (DUF5641)	1.08E-05
PF13837	Myb/SANT-like DNA-binding domain	3.76E-05
PF05729	NACHT domain	4.43E-05
PF18738	DZIP3/ hRUL138-like HEPN	0.000104655
PF03175	DNA polymerase type B, organellar and viral	0.000888925
PF16064	Domain of unknown function (DUF4806)	0.001138042
PF13359	DDE superfamily endonuclease	0.001482117
PF00589	Phage integrase family	0.001616361
PF05380	Pao retrotransposon peptidase	0.001658831
PF02229	Transcriptional Coactivator p15 (PC4)	0.01018659
PF00632	HECT-domain (ubiquitin-transferase)	0.01312261
PF13358	DDE superfamily endonuclease	0.02601291
PF13613	Helix-turn-helix of DDE superfamily endonuclease	0.042545791
PF02892	BED zinc finger	0.062858297
PF15299	Amyotrophic lateral sclerosis 2 chromosomal region candidate gene 8	0.065904043
PF05970	PIF1-like helicase	0.075173466
PF02037	SAP domain	0.083502363
PF01205	Uncharacterized protein family UPF0029	0.096635694
PF00929	Exonuclease	0.130259112
PF00665	Integrase core domain	0.14960198
PF05699	hAT family C-terminal dimerisation region	0.172853021
PF00078	Reverse transcriptase (RNA-dependent DNA polymerase)	0.177783186
PF10551	MULE transposase domain	0.179066499
PF01026	TatD related DNase	0.179066499
PF03732	Retrotransposon gag protein	0.182559425
PF00619	Caspase recruitment domain	0.184465386
PF00856	SET domain	0.185757963
PF17921	Integrase zinc binding domain	0.195160715
PF00653	Inhibitor of Apoptosis domain	0.207778103
PF05485	THAP domain	0.226847046
PF09588	YqaJ-like viral recombinase domain	0.228004009
PF00531	Death domain	0.228518606
PF02338	OTU-like cysteine protease	0.22991394
PF17919	RNase H-like domain found in reverse transcriptase	0.22991394
PF00106	short chain dehydrogenase	0.24880285
PF00046	Homeodomain	0.299109626
PF00098	Zinc knuckle	0.559943628

Supplementary Table S16. Enriched Pfam domains in the non-syntenic region in scaffold 9 (B3 in Supplementary Fig. S13).

Pfam ID	Name	q-value
PF08205	CD80-like C2-set immunoglobulin domain	1.51E-12
PF13927	Immunoglobulin domain	4.13E-07
PF13358	DDE superfamily endonuclease	0.000369767
PF00047	Immunoglobulin domain	0.010109797
PF01498	Transposase	0.010772996
PF05380	Pao retrotransposon peptidase	0.049358054

Supplementary Table S17. Enriched Pfam domains in the non-syntenic region in scaffold 9 (B4 in Supplementary Fig. S13).

Pfam ID	Name	q-value
PF00665	Integrase core domain	8.03E-08
PF17919	RNase H-like domain found in reverse transcriptase	6.17E-07
PF05970	PIF1-like helicase	2.92E-06
PF05729	NACHT domain	3.51E-05
PF18738	DZIP3/ hRUL138-like HEPN	4.38E-05
PF13359	DDE superfamily endonuclease	5.45E-05
PF00078	Reverse transcriptase (RNA-dependent DNA polymerase)	0.00011263
PF17921	Integrase zinc binding domain	0.00036666
PF00077	Retroviral aspartyl protease	0.00194148
PF03175	DNA polymerase type B, organellar and viral	0.00200733
PF13975	gag-polyprotein putative aspartyl protease	0.00228748
PF00098	Zinc knuckle	0.00495659
PF03184	DDE superfamily endonuclease	0.00614491
PF17917	RNase H-like domain found in reverse transcriptase	0.00979369
PF09588	YqaJ-like viral recombinase domain	0.01599518
PF14291	Domain of unknown function (DUF4371)	0.01779658
PF18701	Family of unknown function (DUF5641)	0.02099513
PF03732	Retrotransposon gag protein	0.02856499
PF01026	TatD related DNase	0.02869797
PF02023	SCAN domain	0.02957001
PF04843	Herpesvirus tegument protein, N-terminal conserved region	0.02977466
PF00856	SET domain	0.02977466
PF05699	hAT family C-terminal dimerisation region	0.03016893
PF05380	Pao retrotransposon peptidase	0.03079708
PF00271	Helicase conserved C-terminal domain	0.03098325
PF02513	Spin/Ssty Family	0.0497197
PF00270	DEAD/DEAH box helicase	0.06571808
PF00628	PHD-finger	0.06784887
PF12259	Baculovirus F protein	0.06880796
PF13837	Myb/SANT-like DNA-binding domain	0.07120362
PF05225	helix-turn-helix, Psq domain	0.07120362
PF05485	THAP domain	0.07309997
PF02037	SAP domain	0.08361112
PF04218	CENP-B N-terminal DNA-binding domain	0.08361112
PF04500	FLYWCH zinc finger domain	0.09057366
PF00385	Chromo (CHRromatin Organisation MOdifier) domain	0.09937377
PF03221	Tc5 transposase DNA-binding domain	0.13448332
PF02229	Transcriptional Coactivator p15 (PC4)	0.14125599
PF13894	C2H2-type zinc finger	0.14262524
PF12012	Domain of unknown function (DUF3504)	0.15831634
PF00632	HECT-domain (ubiquitin-transferase)	0.15831634
PF10551	MULE transposase domain	0.19526336
PF13358	DDE superfamily endonuclease	0.21175362
PF00619	Caspase recruitment domain	0.21503872
PF13613	Helix-turn-helix of DDE superfamily endonuclease	0.25807191
PF13472	GDSL-like Lipase/Acylhydrolase family	0.26008872
PF14214	Helitron helicase-like domain at N-terminus	0.26111196
PF14893	PNMA	0.26316874
PF02338	OTU-like cysteine protease	0.27234685
PF00096	Zinc finger, C2H2 type	0.32126208

Supplementary Table S18. The number of proteins with specific functional domains encoded in animal genomes.

Phylum	Class	Species	NACHT	HEPN_DZIP3	NACHT +HEPN_DZIP3	NACHT +RNI-like LRR	NACHT +TPR	NACHT +WD40	NACHT +Death	NACHT +CARD
Mollusca	Bivalvia	*Pinctada fucata* (Haplotype A)	218	103	52	128	5	10	19	1
Mollusca	Bivalvia	*Pinctada fucata* (Haplotype B)	208	98	57	123	4	9	19	1
Mollusca	Bivalvia	*Crassostrea gigas*	15	128	1	1	2	8	1	1
Mollusca	Bivalvia	*Crassostrea virginica*	20	145	2	2	3	9	2	2
Mollusca	Bivalvia	*Bathymodiolus platifrons*	12	138	1	1	2	4	1	1
Mollusca	Bivalvia	*Modiolus philippinarum*	13	145	1	1	2	4	1	1
Mollusca	Bivalvia	*Mizuhopecten yessoensis*	22	59	3	3	4	14	3	3
Mollusca	Bivalvia	*Pecten maximus*	15	41	0	0	3	8	0	0
Mollusca	Gastropoda	*Lottia gigantea*	10	1	1	1	2	5	1	1
Mollusca	Gastropoda	*Haliotis discus*	4	1	1	1	0	0	1	1
Mollusca	Gastropoda	*Gigantopelta aegis*	17	1	1	1	5	10	1	1
Mollusca	Gastropoda	*Pomacea canaliculata*	10	1	1	1	3	3	1	1
Mollusca	Gastropoda	*Aplysia californica*	5	1	1	1	0	2	0	0
Mollusca	Gastropoda	*Biomphalaria glabrata*	12	0	0	0	6	8	0	0
Mollusca	Cephalopoda	*Octopus bimaculoides*	7	0	0	1	2	1	0	0
Mollusca	Cephalopoda	*Octopus sinensis*	7	0	0	1	2	2	0	0
Mollusca	Cephalopoda	*Sepia pharaonis*	2	0	0	0	1	1	0	0
Nemertea	Anopla	*Notospermus geniculatus*	26	1	1	1	4	13	2	2
Phoronida		*Phoronis australis*	21	4	1	1	3	11	5	5
Brachiopoda	Lingulata	*Lingula anatina*	25	27	5	7	1	4	3	3
Annelida	Polychaeta	*Capitella teleta*	107	1	1	60	3	7	56	55
Annelida	Clitellata	*Helobdella robusta*	2	0	0	0	0	0	0	0
Platyhelminthes		*Schistosoma mansoni*	0	0	0	0	0	0	0	0
Platyhelminthes		*Schmidtea mediterranea*	2	1	1	1	0	0	1	1
Nematoda	Chromadorea	*Caenorhabditis elegans*	1	0	0	0	0	1	0	0
Arthropoda	Insecta	*Drosophila melanogaster*	1	0	0	0	0	0	0	0
Arthropoda	Insecta	*Tribolium castaneum*	1	0	0	0	0	0	0	0
Echinoderma	Echinoidea	*Strongylocentrotus purpuratus*	245	0	0	90	8	11	64	0
Chordata	Ascidiacea	*Ciona intestinalis*	27	1	1	13	0	1	0	0
Chordata	Leptocardii	*Branchiostoma floridae*	82	10	7	48	3	8	31	5
Chordata	Actinopterygii	*Danio rerio*	260	0	0	176	0	2	45	9
Chordata	Aves	*Gallus gallus*	7	0	0	6	1	1	3	2
Chordata	Mammalia	*Homo sapiens*	26	1	0	21	0	3	16	3
Cnidaria	Hydrozoa	*Hydra magnipapillata*	24	3	0	0	2	2	2	0
Cnidaria	Anthozoa	*Acropora digitifera*	403	89	30	257	6	7	11	0
Cnidaria	Anthozoa	*Nematostella vectensis*	20	23	7	6	0	1	1	0
Porifera	Demospongiae	*Amphimedon queenslandica*	227	0	0	78	0	2	17	17
Placozoa		*Trichoplax adhaerens*	10	0	0	0	0	0	0	0

Supplementary Table S19. The number of genes encoding conserved Pfam domains combined with NACHT.

Pfam ID	Number of genes in Haplotype A	Number of genes in Haplotype B	Description
PF18738	50	52	DZIP3/ hRUL138-like HEPN
PF13271	10	8	Domain of unknown function (DUF4062)
PF00531	5	5	Death domain
PF15112	7	2	Domain of unknown function (DUF4559)
PF00400	4	4	WD domain, G-beta repeat
PF13424	4	3	Tetratricopeptide repeat
PF13374	1	2	Tetratricopeptide repeat
PF18701	1	1	Family of unknown function (DUF5641)
PF00619	1	1	Caspase recruitment domain
PF00805	1	1	Pentapeptide repeats (8 copies)
PF05731	1	1	TROVE domain
PF12894	1	1	Anaphase-promoting complex subunit 4 WD40 domain
PF17908	1	1	APAF-1 helical domain
PF04300	1	1	F-box associated region
PF00664	1	1	ABC transporter transmembrane region
PF05380	1	1	Pao retrotransposon peptidase
PF00005	1	1	ABC transporter
PF03732	0	1	Retrotransposon gag protein
PF05970	0	1	PIF1-like helicase
PF11715	0	1	Nucleoporin Nup120/160
PF08477	0	1	Ras of Complex, Roc, domain of DAPkinase
PF00041	0	1	Fibronectin type III domain
PF12012	0	1	Domain of unknown function (DUF3504)
PF00059	0	1	Lectin C-type domain
PF09486	1	0	Bacterial type III secretion protein (HrpB7)
PF13432	1	0	Tetratricopeptide repeat
PF17921	0	1	Integrase zinc binding domain
PF13975	0	1	gag-polyprotein putative aspartyl protease
PF00098	0	1	Zinc knuckle
PF07723	0	1	Leucine Rich Repeat

Supplementary Table S20. The number of genes encoding conserved superfamilies combined with NACHT.

ID	Number of genes in Haplotype A	Number of genes in Haplotype B	Description
SSF52047	124	119	RNI-like Leucine-rich repeat
SSF52540	86	86	P-loop containing nucleoside triphosphate hydrolases
SSF47986	19	19	DEATH domain
SSF57997	15	11	Tropomyosin
SSF52058	14	9	L domain-like
SSF58104	16	7	Methyl-accepting chemotaxis protein (MCP) signaling domain
SSF50978	7	5	WD40 repeat-like
SSF50998	6	6	Quinoprotein alcohol dehydrogenase-like
SSF48452	5	4	TPR-like
SSF48371	3	3	ARM repeat
SSF50969	2	2	YVTN repeat-like/Quinoprotein amine dehydrogenase
SSF101908	0	3	Putative isomerase YbhE
SSF56436	1	1	C-type lectin-like
SSF53098	1	1	Ribonuclease H-like
SSF56672	1	1	DNA/RNA polymerases
SSF140864	1	1	TROVE domain-like
SSF141571	1	1	Pentapeptide repeat-like
SSF49785	1	1	Galactose-binding domain-like
SSF49265	1	1	Fibronectin type III
SSF90123	1	1	ABC transporter transmembrane region
SSF57903	0	1	FYVE/PHD zinc finger
SSF63829	0	1	Calcium-dependent phosphotriesterase
SSF57756	0	1	Retrovirus zinc finger-like domains
SSF51004	1	0	C-terminal (heme d1) domain of cytochrome cd1-nitrite reductase
SSF50630	0	1	Acid proteases
SSF69322	1	0	Tricorn protease domain 2

References

1. Takeuchi, T., Kawashima, T., Koyanagi, R., et al. 2012, Draft genome of the pearl oyster *Pinctada fucata*: a platform for understanding bivalve biology. *DNA Res.*, **19**, 117–30.

2. Takeuchi, T., Koyanagi, R., Gyoja, F., et al. 2016, Bivalve-specific gene expansion in the pearl oyster genome: implications of adaptation to a sessile lifestyle. *Zool. Lett.*, **2**, 3.

3. Bolger, A. M., Lohse, M., and Usadel, B. 2014, Trimmomatic: A flexible trimmer for Illumina Sequence Data. *Bioinformatics*, **30**, 2114–20.

4. Chin, C.-S., Peluso, P., Sedlazeck, F. J., et al. 2016, Phased diploid genome assembly with single-molecule real-time sequencing. *Nature Methods*, **13**, 1050–4.

5. Zimin, A. V., Marçais, G., Puiu, D., Roberts, M., Salzberg, S. L., and Yorke, J. A. 2013, The MaSuRCA genome assembler. *Bioinformatics*, **29**, 2669–77.

6. Koren, S., Walenz, B. P., Berlin, K., Miller, J. R., Bergman, N. H., and Phillippy, A. M. 2017, Canu: scalable and accurate long-read assembly via adaptive k-mer weighting and repeat separation. *Genome Res.*, **27**, 722–36.

7. Li, H. 2013, May 26, Aligning sequence reads, clone sequences and assembly contigs with BWA-MEM.

8. Walker, B. J., Abeel, T., Shea, T., et al. 2014, Pilon: An Integrated Tool for Comprehensive Microbial Variant Detection and Genome Assembly Improvement. *PLOS ONE*, **9**, e112963.

9. Huang, S., Kang, M., and Xu, A. 2017, HaploMerger2: rebuilding both haploid sub-assemblies from high-heterozygosity diploid genome assembly. *Bioinformatics*, **33**, 2577–9.

10. Zheng, G. X. Y., Lau, B. T., Schnall-Levin, M., et al. 2016, Haplotyping germline and cancer genomes with high-throughput linked-read sequencing. *Nat Biotechnol*, **34**, 303–11.

11. Putnam, N. H., O'Connell, B. L., Stites, J. C., et al. 2016, Chromosome-scale shotgun assembly using an in vitro method for long-range linkage. *Genome Res.*, **26**, 342–50.

12. Marçais, G., and Kingsford, C. 2011, A fast, lock-free approach for efficient parallel counting of occurrences of k-mers. *Bioinformatics*, **27**, 764–70.

13. Vurture, G. W., Sedlazeck, F. J., Nattestad, M., et al. 2017, GenomeScope: fast reference-free genome profiling from short reads. *Bioinformatics*, **33**, 2202–4.

14. Li, H., and Durbin, R. 2009, Fast and accurate short read alignment with Burrows–Wheeler transform. *Bioinformatics*, **25**, 1754–60.

15. Li, H. 2011, A statistical framework for SNP calling, mutation discovery, association mapping and population genetical parameter estimation from sequencing data. *Bioinformatics*, **27**, 2987–93.

16. Kurtz, S., Phillippy, A., Delcher, A. L., et al. 2004, Versatile and open software for comparing large genomes. *Genome Biology*, **5**, R12.
